# Simulating the effects of local adaptation and life history on the ability of plants to track climate shifts

**DOI:** 10.1093/aobpla/plaa008

**Published:** 2020-02-14

**Authors:** Emily V Moran

**Affiliations:** Department of Life and Environmental Sciences, University of California Merced, Merced, CA, USA

**Keywords:** Climate change, dispersal, life history, local adaptation, range shift, simulation

## Abstract

Many studies have examined the impact of dispersal on local adaptation, but much less attention has been paid to how local adaptation influences range shifts. The aim of this study was to test how local adaptation might affect climate-driven range shifts in plants, and if this might differ between plants with different life histories. Simulated range shift dynamics were compared for hypothetical annual, perennial and tree species, each comprised of either one plastic genotype or six locally adapted genotypes. The landscape consists of shifting climate bands made up of 20 × 20 m patches containing multiple individuals. Effects of seed dispersal, breadth of the plastic species’ tolerance, steepness of the climate gradient and rate of the climate shift are also examined. Local adaptation increased the equilibrium range size and aided range shifts by boosting fitness near range edges. However, when the rate of climate change was doubled on a steep gradient, locally adapted trees exhibited a higher percent loss of range during the climate shift. The plastic annual species with short dispersal was unable to recover its range size even after the climate stabilized, while the locally adapted annuals tracked climate change well. The results suggest that in most situations local adaptation and longer dispersal distances will be advantageous, though not necessarily sufficient, for tracking suitable climates. However, local adaptation might put species with long generation times at greater risk when climate shifts are very rapid. If confirmed by empirical tests, these results suggest that identifying variation between species in how fitness varies along climate gradients and in these key demographic rates might aid in prioritizing management actions.

## Teaser

This paper uses a simulation model to explore how local adaptation in plants may shape responses to climate shifts. When local adaptation improved survival and reproduction at range edges, this increased the area occupied when climate is stable and usually improved tracking of climate shifts. Local adaptation yielded the biggest benefit for low-dispersal annual species. However, the area occupied by the species can still lag behind climate change even when the period of climate change and the distance to reach newly suitable areas are both short. This is especially true of trees; locally adapted trees sometimes exhibited greater lags.

## Introduction

Considerable research effort has focused on the impact of gene flow on local adaptation (Kirkpatrick and Barton 1997; Lenormand 2002; Holt *et al.* 2004; Bridle and Vines 2006; Garant *et al.* 2007; Kimbrell and Holt 2007; Kawecki 2008; Räsänen and Hendry 2008; Bell and Gonzales 2009; Bridle *et al.* 2009, 2010; Sexton *et al.* 2009, 2011; Dawson *et al.* 2010; North *et al.* 2010; Kremer *et al.* 2012; Polechová and Barton 2015). Less attention has been paid to how local adaptation affects the potential for range shifts under climate change. In addition, it is not clear whether effects would be the same across life history types.

Researchers have defined and tested the degree of local adaptation in plants in two ways. The first approach, generally based on a small number of reciprocal transplants, classifies populations as locally adapted if they either perform better in their home site than in other sites or if, within a site, local genotypes perform better than foreign ones (Kawecki and Ebert 2004). The second approach, used most commonly in provenance studies of trees in which many seed sources are planted in multiple common gardens (not including all source sites), defines a population as being locally adapted if it performs best in sites that are more environmentally similar to its home (Aitken and Whitlock 2013; Browne *et al.* 2019). This sense is commonly used in studies focusing on performance along environmental gradients (Angert *et al.* 2011; Leites *et al.* 2012; Moran *et al.* 2017), variation in traits or specific genetic markers relative to null expectations (Keller *et al.* 2011; Csilléry *et al.* 2014) or responses to changing environmental conditions (Atkins and Travis 2010; Bocedi *et al.* 2013; Browne *et al.* 2019). This definition has the benefit of specifying *what* a species is adapted to, and is therefore how ‘locally adapted’ will be used here.

Local adaptation is common but not universal in plants (Leimu and Fischer 2008; Hereford 2009; Browne *et al.* 2019) and results from the interplay of gene flow with natural selection (Garant *et al.* 2007; Kawecki 2008; Räsänen and Hendry 2008; Sexton *et al.* 2009). Gene flow can increase local genetic variation and population size, which boost adaptive potential (Holt *et al.* 2004; Kimbrell and Holt 2007; North *et al.* 2010; Kremer *et al.* 2012), but excessive immigration from environmentally distinct areas may also ‘swamp’ local adaptation (Kirkpatrick and Barton 1997; Lenormand 2002; Bridle and Vines 2006; Kawecki 2008). Strong selection can, however, create local adaptation in the presence of high gene flow (Gonzalo-Turpin and Hazard 2009; Csilléry *et al.* 2014; Eckert *et al.* 2015; Moran *et al.* 2017), which can result in a pattern of ‘isolation by environment’ (IBE) at loci relevant for fitness but little differentiation at neutral loci (Sexton *et al.* 2014).

Several studies have examined how the existence of locally adapted subdivisions within widespread species may affect habitat suitability as climate changes. Most have focused on trees, as provenance experiments in the 20th century yielded much information on local adaptation to climate (Leites *et al.* 2012). Taking population subdivisions into account often results in less negative predictions of climate change impacts on species ranges (Garzon *et al.* 2011; Oney *et al.* 2013; Maguire *et al.* 2018). Few studies have examined this in herbaceous plants. One exception was a study of *Mimulus cardinalis* that found that population-level mechanistic models predicted greater range size following climate change than a mechanistic model fit to species-level average temperature responses (Angert *et al.* 2011). However, most such studies have not examined the species’ ability to fill suitable habitat through dispersal.

**Figure 1.**
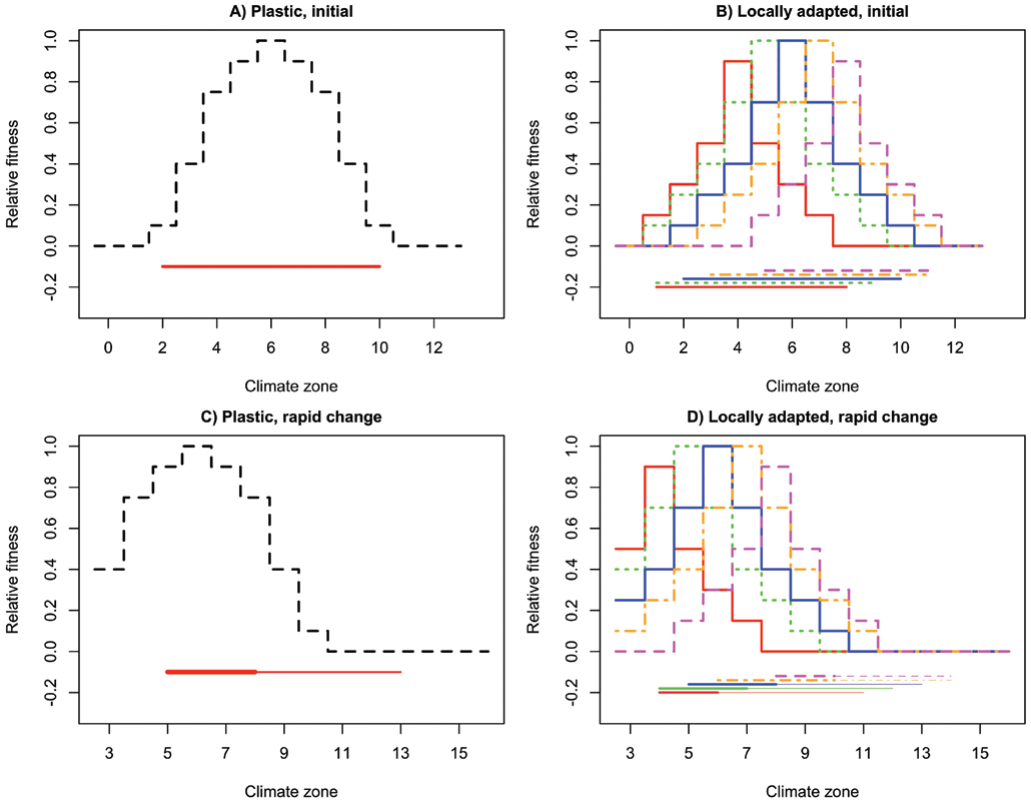
(A) Dashed black curve depicts fitness across climate zones for a hypothetical plastic species, horizontal red line the area that species might occupy on the landscape. (B) Coloured curves depict fitness across climate zones for each of five populations in a locally adapted species. Horizontal coloured lines depict area of landscape each population could occupy in the absence of competition from other populations. (C) Dashed black curve depicts fitness across climate zones for plastic species after a period of rapid climate change. Horizontal red line depicts the original range, with areas where fitness increases drawn thick and areas where fitness decreases drawn thin. (D) Coloured curves depict fitness across climate zones for each of five populations after rapid climate change. Horizontal coloured lines depict the original range, with areas where fitness increases drawn thick and areas where fitness decreases drawn thin.

Local adaptation may reduce fitness differences between populations across the range, though such specialization can have costs. For instance, in the invasive plant *Lythrum salicaria* northern populations evolved earlier flowering, which increased their reproductive output there, but associated decreases in vegetative growth decreased the fitness of these genotypes in the south (Colautti and Barrett 2013). Figure 1 depicts two hypothetical species, one made up of a single population or genotype with wide climatic tolerances (Fig. 1A), and one made up of several populations or genotypes, each with narrower climatic tolerances (Fig. 1B). If the climate gradient shifts, species 1 will likely see a greater change in habitat suitability overall (Fig. 1C and D). However, each narrowly adapted population/genotype of species 2, if it stays in place and climate change continues, will more rapidly find itself in conditions to which it is maladapted. Equalization of fitness across the range is important, as the furthest-forward individuals are the most likely source for seeds dispersing beyond the current range edge (Clark 1998; Clark *et al.* 2001). Conversely, a lack of seed produced near the range edge could slow expansion (Kroiss and HilleRisLambers 2015).

Simulation model studies disagree about the impact of local adaptation on range shift ability. Some of this disagreement stems from details of how the models are constructed, particularly what assumptions they make about the structure of the landscape and population. For instance, Moran and Ormond (2015), Bocedi *et al.* (2013) and Atkins and Travis (2010) all considered species spreading along a linear climate gradient that shifts by a fixed amount each year, in which climate effects on fitness are determined by the genotype at a diploid locus. Atkins and Travis found that cold-adapted genotypes tended to block the spread of warm-adapted genotypes, but this is likely because only one individual could occupy each patch/cell in the landscape, mortality was independent of climate and the landscape was small (200 × 200 patches). Models that allow multiple individuals to occupy a patch and that considered larger landscapes have not found this effect (Bocedi *et al.* 2013; Moran and Ormond 2015). Bocedi *et al.* (2013) found that cold-adapted alleles could ‘surf’ backwards and displace warm-adapted alleles, and that ranges could become disjointed, with a loss of central alleles. These patterns may have been affected by the fact that only 20 % of the landscape was treated as suitable habitat, making spread of all genotypes more difficult. The Moran and Ormond model (2015) treated habitat as continuous and found that local adaptation in a low-elevation species aided spread to higher-elevation habitats, while local adaptation of the high-elevation species slowed spread of the low-elevation species.

It is possible that life history and dispersal characteristics might also influence the effect of local adaptation on range shifts. Species with shorter generation times could exhibit more rapid responses to changing conditions. However, plant species that take longer to mature often have greater height and larger seed crops, which increases the probability of long-distance dispersal. In an analysis of 80 plant species, we found that groups with very different generation times had similar predicted average spread rates (around 5.3 m per year), with higher seed production and dispersal in long-lived plants more than balancing out their longer lifespans (Lustenhower *et al.* 2017). There was substantial variation within life history groups due to variation in fecundity and dispersal. On the other hand, species with longer lifespans and generation times might exhibit substantial lags in spread or adaptation as the rate of change increases (Kuparinen *et al.* 2010). However, no previous simulation or empirical study has explicitly examined how life history and local adaptation may interact to affect range shifts under climate change.

In this study, I compare simulations of three basic plant life history types: a wind-pollinated tree with moderately shade-intolerant seedlings, a clump-forming perennial and an annual wildflower. Dispersal distances tend to be positively affected by plant height, and maximum seed crop by overall size, and so are assumed to increase from annual to perennial to tree (Herrera 1991; Dodd and Silvertown 2000; Nathan *et al.* 2011; Lustenhower *et al.* 2017), but I consider several values for seed dispersal parameters that overlap across types. Each life history type may consist of either a single broadly adapted genotype (‘plastic’) or several narrowly adapted genotypes that differ in abundance across the elevation gradient (‘locally adapted’). It should be noted that none of the simulated ‘species’ are meant to represent a single real plant species, or the classes of ‘annual’, ‘perennial’ and ‘tree’ as a whole. Rather, in recognition of the fact that features like dispersal distance and time to maturity do not vary independently (Lustenhower *et al.* 2017), they are meant to represent plausible combinations of demographic rates within these categories.

In part 1, I compare range shift responses along an elevation gradient during and after a short period of moderately rapid climate change for plastic or locally adapted versions of each of these three types. In part 2, I test how the rate of decline in fitness at the range margins affects spread independent of number of genotypes. This is done by making the maximum fitness of the plastic perennial genotype across the range match that of the locally adapted perennial species overall. In part 3, I test the cumulative impact of annual climate effects on range size for species that live for >1 year. To do this, I weaken the impact of climate on annual demographic rates of the perennial species such that the lifetime effects more closely resemble those of the annual species. Finally, in part 4, I test whether the long generation times of trees change the effects seen in part 1 if climate change is more rapid or climate bands are broader.

I hypothesized that:

1) If a species is locally adapted such that close-to-maximum fitness is maintained near the climatic limits of the species, this will result in a wider equilibrium range and less lag in responding to a climate shift than in an equivalent plastic species with fitness that declines more gradually.
2) If the plastic species has a single population/genotype with the same maximum fitness across environments as all the subpopulations/genotypes of the locally adapted species, it will have a broader equilibrium range and lower lag in climate response than either the narrowly plastic or locally adapted species.
3) Species with high seed movement and/or short generation times will exhibit lower lags in range shifts.
4) If annual survival or reproduction is affected equally by climate for all life histories, the species with the longer generation times will exhibit a narrower geographic range, as multiple years of selection will eliminate them from marginal habitats before or soon after they begin to reproduce.

## Methods

This model is based on Moran and Ormond (2015). The main changes are to the dispersal calculations, to make them more easily adjustable to different grid sizes, and the inclusion of life history options other than trees. The landscape is also smaller and finer-grained and there are six genotypes per species instead of three. The finer-grained landscape was introduced because a 50 m × 50 m patch would be too large to capture the dynamics of plants that disperse 20 m or less on average. Similarly, including three alleles that result in six genotypes results in a subtler and hopefully more realistic species response to climate gradients than the two allele/three genotype model.

### Demographic rates

#### Size classes

The annuals have two life stages, seed and adult. The perennials have four size classes in addition to seed: seedlings plus small, medium and large adults. Perennial seedlings are non-reproductive. The trees have an additional non-reproductive size category (saplings) between seedlings and small adults. In both perennials and trees, larger adults produce more seed and pollen (Table 1).

**Table 1.** Demographic parameters. ‘K’ indicates thousands. Maximum numbers of seed or pollen and maximum survival and transition rates are given for each size category, where applicable.

	Annual	Perennial	Tree
Mean seed dispersal (m)	1, 20 or 50	20 or 80	40 or 80
Mean pollen dispersal (m)	60	80	180
Maximum germination rate	0.3	0.45	0.6
Maximum seeds/pollen	300/8K	0/0, 150/800, 1K/10K, 5K/100K	0/0, 0/0, 150/800, 1K/10K, 5K/100K
Maximum survival	0	0.3, 0.5, 0.65, 0.7	0.6, 0.85, 0.96, 0.98, 0.99
Maximum transition rate	0	0.3, 0.15, 0.1, 0	0.15, 0.11, 0.06, 0.02, 0
Individual size	NA	0.004, 0.06, 0.25, 2 (m^2^)	0.0001, 0.003, 0.07, 0.14, 0.5 (m^2^ BA)
Competitive effects on germination	NA	0.003 × total area occupied	0.08 × total BA
Competitive effects on survival or transition	Based on number of individuals	0.005, 0.003, 0.001 or 0.0005 × total area of larger individuals	0.1, 0.07, 0.02, 0.01 or 0 × total BA of larger individuals
Standard equilibration period	20 years	100 years	200 years

#### Dispersal

Dispersal in all species follows a fat-tailed 2Dt kernel (Clark *et al.* 1999). I compared two or three different average seed dispersal distances for each species (Table 1). In a previous study, we found that seed dispersal distances varied widely within life history classes but tended to be higher for trees (mean 4.2–100 m) than for perennials (mean 0.2–33.3 m) or annuals (mean 0.1–20 m) (Lustenhower *et al.* 2017). However, due to various constraints and what could be included in the spread rate model, that study assumed negative exponential dispersal kernels. In reality, seed dispersal kernels tend to have more long-distance events that increase the mean than expected under normal or exponential distributions (Clark *et al.* 2005; Cousens *et al.* 2008). Therefore, I included a 50 m average version for the annual, and an 80 m average version for the perennial, since I was envisioning the latter as a tall, wind-dispersed clump-former such as *Solidago* or *Asclepias*. The tree averages were put near but not beyond the top of the range estimated for exponential kernels, as these hypothetical species were meant to represent a moderately heavy-seeded type such as *Pinus* or *Quercus*. 2Dt dispersal kernels estimated for *Quercus rubra* based on genetic markers indicated a mean dispersal distance of 15 m at one site and 125 m at another (Moran and Clark 2012).

 Data on pollen dispersal distances across different plant life history classes are sparse. For simplicity, I assumed that the trees are wind-pollinated, with an average dispersal distance of 180 m, while the herbaceous plants are pollinated by insects with a moderately long flight distance: 80 m for the taller perennial, 60 m for the shorter and less visible annual (Table 1). Because of the fat tail of the probability distribution many of the seeds or pollen grains stay in their patch of origin, but some disperse much further than the mean distance **[see****Supporting Information—Fig. S1.1****]**. Dispersal calculations are explained in **Supporting Information—Appendix S1**.

#### Initialization and sequence of events

The initial numbers and sizes of each genotype are given in **Supporting Information—Appendix S1**. These numbers are arbitrary but large enough to avoid stochastic extinction and each genotype is distributed across the landscape somewhat more broadly than the expected equilibrium distribution. Annual plants were initiated as seeds, perennials as small adults and trees as saplings. In each simulation, there is an initialization period to allow the species to equilibrate with the climate that corresponds roughly to 20 generations (Table 1).

Each year of the simulation starts with the germination of seeds (if present). Non-germinating seeds are removed, meaning there is no persistent seed bank. Survival of each size class for the year is then calculated, followed by pollen production and dispersal, seed production and dispersal, and transition to larger size classes.

### Demographic rates given climate and competition

Demographic rates for all three life history types are given in Table 1. Again, it should be noted that none of the simulated ‘species’ are meant to represent a ‘type’ rather than single real plant species. Like dispersal, fecundity (seeds per year) differs substantially between real species within a particular life history class (Lustenhower *et al.* 2017). Because the simulated annual species was envisioned as a relatively small-bodied plant, its annual fecundity was set closer to the low end of the observed range (3–82 000). The *Solidago* and *Asclepias* species included in this previous analysis had estimated mean fecundities of 450 and 600 seeds per year, but *Solidago canadensis* (formerly *altissima*) can produce over 20 000 seeds per year (Meyer and Schmid 1999), so the maximum fecundity for the simulated perennial species was set at an intermediate value of 5000. For *Pinus* and *Quercus* annual fecundities included in the previous study ranged from 216 to 12 000, so a maximum value of 5000 was considered reasonable for the simulated tree species as well. Annuals by definition mature within a year and then die. For the other species, mortality and growth rates were set such that average time to maturity fell within the range observed in the previous study for perennials (1–17 years) and trees (9–40 years) (Lustenhower *et al.* 2017).

Production of seed and pollen is affected by climate, with fecundity at one or more climate steps from the optimum being a fraction of the maximum. Thus:

$$\begin{array}{l} Seed/Pollen\;\;\;=\;\;\;Max.Seed/Pollen\;\;\;\times \;\;\;ClimEff[C.steps] \end{array}$$

where ClimEff[C.steps] is the climate effect corresponding to a certain number of steps away from the optimum.

For all life history classes, the number of individuals germinating, surviving or transitioning for each genotype in each size class in each patch is generated by drawing from a Bernoulli distribution, such that:

$$\begin{array}{l} N_{1}∼Bern(N_{0},\;\;\;Rate) \end{array}$$

where *N*_0_ is the number of individuals before the event.

All demographic rates in the tree and perennial are affected by both climate and a competitive effect (CompEff) that depends either on the basal area (BA) of larger trees or the surface area occupied by larger perennials in the patch (TA) and the size of the target individual (Table 1). Thus:

$$\begin{array}{l} Rate=\;\;\;Max.Rate\;\;\;(ClimEff[C.steps]-(CompEff\;\;\;\times \;\;\;CA)) \end{array}$$

where Max.Rate is the maximum possible rate and CA is the surface or BA of competitors in the patch.

In annuals, germination is affected only by climate, as no plants exist on the landscape prior to this step:

$$\begin{array}{l} Germ.rate_{A}\;\;\;=\;\;\;Max.Germ\;\;\;\times \;\;\;ClimEff[C.steps] \end{array}$$

However, because competitive interactions might become important as annuals grow, if the number of seedlings in a patch exceeds 7000, the probability of survival to reproduction = 7000/(initial seedling density). Annuals live only 1 year and do not transition.

**Figure 2.**
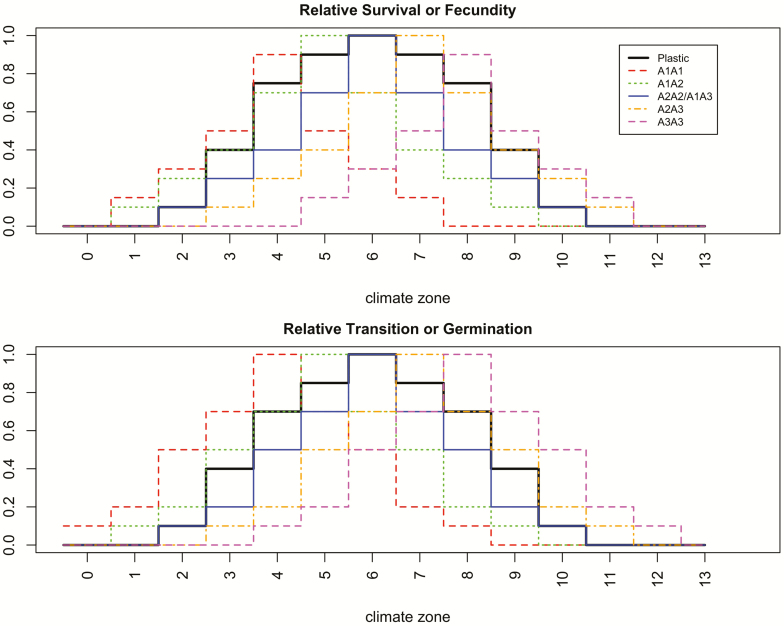
Demographic rates relative to maximum in each climate zone for either the single plastic genotype (dark black line), or individual genotypes/populations in the variable species (coloured lines).

In all cases, if the calculated rate is less than zero, the rate is set to zero. Maximum demographic rates and competitive effects on demographic rates are given in Table 1. Standard climate effects on demographic rates for each genotype are illustrated in Fig. 2 and described further in **Supporting Information—Appendix S1**. Note that in the species with multiple genotypes (the initially locally adapted species), each genotype has a different optimum climate, with the optima for heterozygotes being intermediate between the optima for the homozygotes. For the widely plastic single genotype (part 2), the response curve traces the maximum species-level fitness of the locally adapted species **[see****Supporting Information—Fig. S1.2****]**. In part 3, to make the lifetime fitness effects of climate more similar for perennials and annuals, I reduced the negative impacts of each step away from the optimum climate by half **[see****Supporting Information—Fig. S1.2****]**.

### Landscape and climate

In a mountainous landscape, a shift in elevation of 1000 m (3280 ft) often represents a major shift in vegetation. For instance, in Sequoia National Park, plots at 1500 m in elevation contain a diverse mix of white fir, incense cedar, sugar and ponderosa pines, and black oak, while at around 2500 m they are dominated by red fir and lodgepole or western white pine (Moran *et al.* 2019). A shift of 1 km along the surface of a mountain slope can represent a shift of 745–913 m in elevation for slopes of 20–40 degrees. The ‘standard’ landscape therefore consisted of a grid 1 km wide × 2 km representing a ‘slice’ of a species’ range an elevation gradient, with climate bands 200 m wide ranging from warmer at the left to cooler at the right. The 2 km low-high axis thus represents a 1490 to 1826 m change in elevation. The exact relationship between temperature and elevation can vary, and this landscape is not meant to replicate any real landscape. However, for reference, an elevation gradient of this size the Sequoia National Park example would represent at least a 7.3 °C difference in mean annual temperature and a 10.6 °C difference in July maximum temperature (Moran *et al.* 2019). Each patch within the landscape represents a 20 × 20 m area and can accommodate multiple individuals.

After the initialization period, the climate bands shift at a rate of 20 m (one patch width) per year for 20 years, such that the range of available climates ranges from 10 (warmest) to 1 (coldest) at the beginning and from 12 to 3 at the end, the two coldest climates disappearing entirely. This rate is close to the mean rates estimated by Loarie *et al.* (2009) for many montane environments under an A1B emission scenario. The populations were then allowed to adjust to the new climate gradient for a length of time equal to the initial equilibration period. The number of patches occupied by adults of each genotype and by the species as a whole were recorded at the end of the 20-year climate change period and at the end of the re-equilibration period, and compared to occupancy at the start. Actual contemporary climate change is of course projected continue at these rates for far beyond 20 years (Loarie *et al.* 2009; IPCC 2013). The limited time period was chosen so that loss in range would be mostly due to lags in climate tracking rather than loss of suitable habitat from the landscape; over longer time periods, both sources of range loss would be more severe.

Variations used include:

- Allowing 10× as long for restabilization when the species appeared far from equilibrium with climate after the standard restabilization period.
- Increasing the rate of the climate shift from 20 to 40 m per year to assess the impact on trees (the category with the longest generation times).
- A landscape in which the coldest climate bands was three times wider. This was used where the species would otherwise lose total suitable habitat, and allowed me to check whether a reduction in occupied range was due to the loss of suitable habitat or to failure to fill suitable habitat.
- A much longer landscape (8 km) with a shallower gradient, in which all climate bands are four times as wide as in the standard landscape. The climate shifted at a rate of four patch widths (80 m) per year so that the colder climate bands disappear at the same rate as in the standard scenario. This was used to assess the effect of a less steep gradient when seeds have to travel farther to reach a cooler climate.

## Results

### Part 1—Standard model

#### Initial occupancy

**Figure 3.**
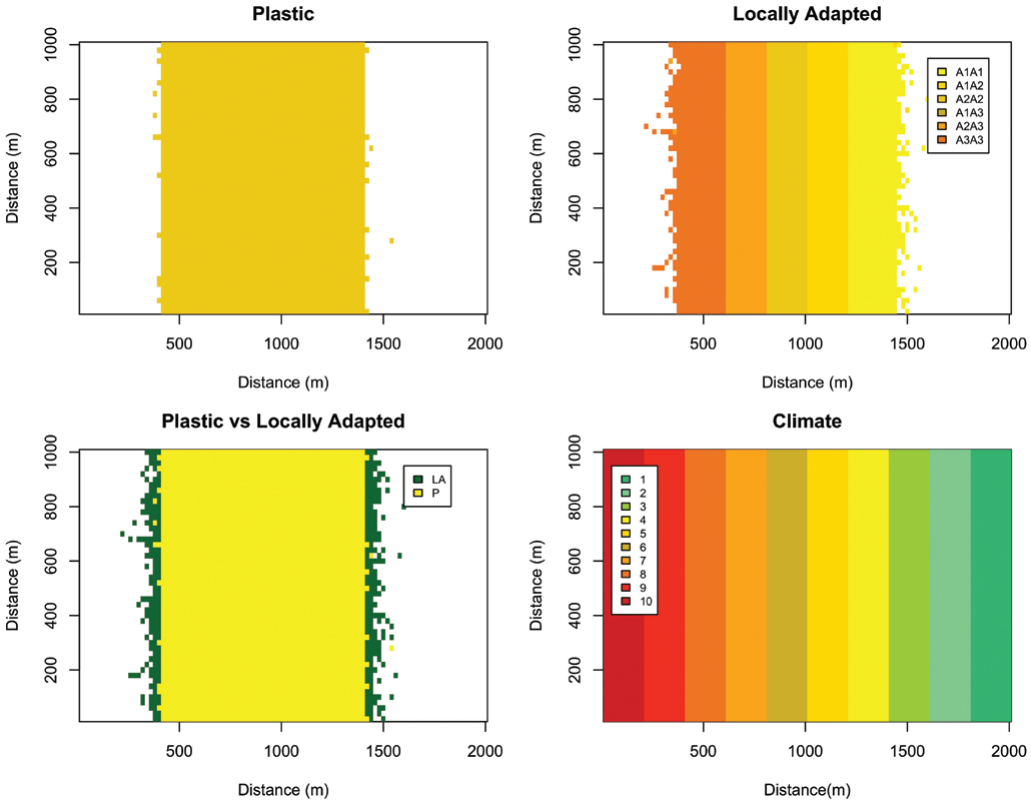
Example pre-shift geographic range for a tree with 40 m average dispersal. The top two panels show the patches occupied by the plastic species (single genotype, optimum climate = 6), and the locally adapted species (colour corresponding to most common genotype and its favoured climate). The plastic vs. locally adapted panel illustrates that the locally adapted species (LA) has a slightly wider geographic range than the plastic species (P).

**Figure 4.**
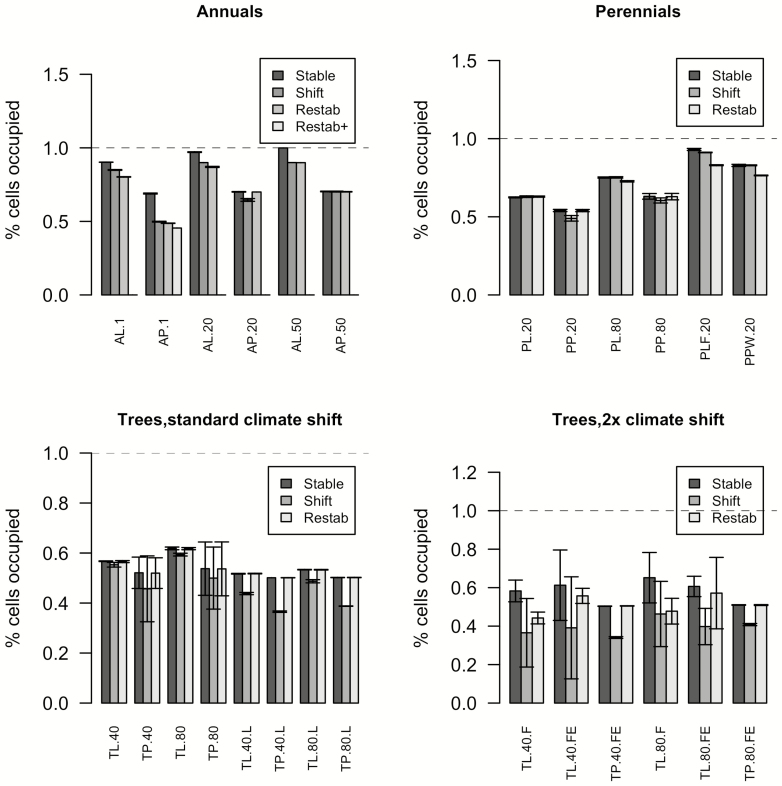
Percent of landscape occupied after initial stabilization period (‘Stable’), at the end of the period of climate change (‘shift’) and after the restabilization period (‘Restab’). AL = annual, locally adapted; AP = annual, plastic; PL = perennial, locally adapted; PP = perennial, plastic; TL = tree, locally adapted; TP = tree, plastic. Numbers following these codes indicate average dispersal distance in meters. (Top left panel) For AP.1, the plastic annual with 1 m dispersal, a restabilization period 10× as long was tested in one run (‘Restab+’). (Top right panel) Variants for perennial species include PLF (a locally adapted with half the annual fitness impacts of climate) and PPW (widely plastic). (Bottom left) Trees on standard landscape or on landscape where climate bands are 4× as wide (.L variants), in which the two coldest climate bands disappear by the end of the simulation. (Bottom right) Trees exposed to a faster climate shift on the steeper gradient landscape with either the standard setup (.F variants) or an extended landscape that ensures suitable climates do not run off the edge (.FE variants). Error bars show SD over four model runs.

During the establishment period, the abundance of the species across the landscape changes and genotypes sort themselves into bands corresponding to their areas of highest fitness (Fig. 3). Note that the populations in these bands would thus meet either the ‘home vs. away’ or ‘local vs. foreign’ definitions of being locally adapted (Kawecki and Ebert 2004). For example, if one examines Fig. 2, one can see that if the most common climate zone 5 genotype (A_1_A_2_) were moved to zone 7, it would have lower survival, fecundity, germination and growth than the local genotype (A_2_A_3_), and vice versa. As hypothesized, occupancy of the landscape under the initial climate conditions was greater for the locally adapted species than the plastic one, due to higher fitness near the range edges (Figs 3 and 4). Initial occupancy was also higher for longer dispersal distances (Fig. 4). Due to the cumulative impacts of climate on yearly demographic rates, the geographical distributions are smaller for longer-lived species (Fig. 4).

#### Lags during and after climate shift

As the climate gradient shifts, most species exhibit a reduction in range occupancy due to extinctions at the trailing edge, lags at the leading edge or both (Fig. 4; **see****Supporting Information—Table S2.1**). This loss was highest for the plastic annual species with a mean seed dispersal distance of 1 m, which lost 27.8 % of its range due to increasing unsuitability of the ‘rear edge’ coupled with a failure to colonize newly suitable territory. This species never extended its range during the simulation, even when 200 years were allowed for restabilization. By contrast, the low-dispersal but locally adapted annual species tracked the climate shift well due to some individuals already being present in the areas that were becoming more suitable. In perennials and trees the low-dispersal plastic species also experienced the greatest lags during the climate shift: 9.3 and 4 % losses for the perennial and the tree, respectively, compared to a 0.7 % gain and 2.5 % loss for their locally adapted counterparts. However, their seed dispersal distances were long enough to make up this gap after climate stabilized. Similarly, the tree with 80 m dispersal exhibited less of a lag when it was locally adapted. The locally adapted annuals with 50 m dispersal and the locally adapted perennials all tracked climate change closely. Occupancy was more variable for plastic than for locally adapted tree species (Fig. 4). After restabilization, species that had very broad initial ranges experienced some range loss due to the loss of climates 1 and 2. All other species except for the short-dispersing plastic annual established a post-climate-change range of similar size to their initial range.

### Part 2—Effects of equalizing range-margin fitness for plastic and locally adapted species

The widely plastic perennial species was able to occupy more territory initially and track climate shifts more closely than either narrowly plastic or locally adapted species with the same dispersal distance. In Fig. 4, this can be seen by comparing PPW.20 to PP.20 and PL.20. This seems to be because it combines the benefits of higher fitness near the range edge with broad climate tolerances for all individuals.

### Part 3—Effects of reducing climate effects on annual demographic rates

Reducing by half the negative effects on demographic rates of climatic mismatch on the perennial species resulted in behaviour that more closely resembled the annual species with the same dispersal distance. This can be seen in Fig. 3 by comparing PLF.20 to PL.20 and AL.20. Initial occupancy was 4637.3 patches (vs. 4854.5 for the annual), with 1.8 % of this initial range being lost during the climate shift (annual lost 7.3 %) and 10.54 % lost after restabilization (annual lost 10.4 %). The initial range was smaller for the perennial than the annual, most likely because the proportion of perennials that reach reproductive age is still smaller. The lag was smaller for the perennial, most likely because fecundity is higher for perennials.

### Part 4—Range shifts with different temporal and spatial scales

When the rate of climate change was doubled, climates 1–4 disappear and the trees lost a large portion of their potential range. This resulted in a reduction in range size post-climate change of 23–26 %. However, when the initial width of the coldest climate band is adjusted so that only climates 1 and 2 are lost, all trees re-equilibrated with the new climate gradient within 200 years. The lags increased substantially with more rapid climate change (Fig. 4; **see****Supporting Information—Table S2.1**). Although the plastic species still had a smaller equilibrium range size than the locally adapted species, it lost less of this original range during the more rapid climate shift: 42.42 % vs. 32.36 % for the 40 m disperser, and 31.94 % vs. 19.97 % for the 80 m disperser. In the locally adapted species, the narrowly warm-adapted genotype at the rear edge lost more habitat patches. The narrowly cold-adapted genotype at the leading edge was slow to respond. In the locally adapted 40 m dispersal scenario few patches at the end of the climate shift contain the A1 allele (at frequencies <50 %), indicating that this cold-tolerant allele was nearly lost. In a reverse of part 1, occupancy was more variable for the locally adapted trees.

When these trees are given a climate gradient in which each climate band is four times as wide, trees occupy a lower proportion of the total landscape than in the standard scenario, likely because fewer seeds reach marginal areas and form sink populations. The lag in climate tracking is greater than in the standard scenario, but less extreme than for the scenario with doubled range shift speed (Fig. 4; **see****Supporting Information—Table S2.1**). The lag is greater for the plastic species than the locally adapted one whether the trees have 40 m dispersal (27.07 % vs. 15.34 % loss) or 80 m dispersal (22.77 % vs. 8.7 % loss). As with the other scenarios, the trees re-equilibrated with the new climate gradient within 200 years.

## Discussion

The results of this analysis suggest that local adaptation is likely to be more of a help than a hindrance to plant species in tracking climate shifts, at if locally adapted species usually have higher reproductive success at the leading range edge than species that are less locally adapted. Local adaptation nearly always increased equilibrium range size and the ability to track climate shifts across life history types. This was most notable in the case of the annual with 1 m average dispersal; the locally adapted species was able to occupy newly suitable territory, while the plastic one was not. The negative effects of local adaptation seen in some previous models are likely due to the structure of the simulated landscape. For instance, a small landscape with single-occupancy patches (Atkins and Travis 2010) can more easily lead to established genotypes blocking the spread of warm-adapted ones. However, if the landscape is highly fragmented, this blocking effect could potentially be seen in a larger landscape.

The one scenario in which this simulation found a negative effect of local adaptation was in trees when the climate shift over 20 years (~1 tree generation) was 10–20 times greater than the average seed dispersal distance. In this scenario, climate change ‘outran’ narrowly adapted genotypes before they could spread. Interestingly, although the rates of climate change in the ‘shallow gradient’ scenario were twice that of the rapid shift scenario, locally adapted species still did better. This may be because the larger population sizes within each band provided more seeds, aiding the spread of all genotypes. However, if any of these climate change scenarios persisted for multiple tree generations—as they are expected to do in reality—the tree populations would likely be badly impacted due to cumulative lags in response combined with loss of potentially suitable habitat.

Contrary to my hypothesis, shorter generation times did not always help track climate shifts. Among plastic species with similar dispersal ability, lags were greater for those with longer generation times. However, among locally adapted species, the perennial experienced no lag while the annual and the tree both did. This may be because the perennial always had greater fecundity than the annual and a shorter generation time than the tree. This ‘perennial advantage’ would not necessarily hold consistently in the real world; as noted in the methods all such plant life history categories contain considerable variation in dispersal ability and fecundity, and generation time in perennials can vary from 1 year to several decades (Lustenhower *et al.* 2017).

How much these results say about relative range shift potential in the real world will depend on how well the assumptions of the model capture the conditions under which plants are spreading in four regards: (i) the qualities of the landscape and climate gradient and how individual plants fill it, (ii) how well the treatment of genotypes captures the process of gene flow and local adaptation, (iii) how local adaptation influences fitness near range edges and (iv) how far seed and pollen disperse.

As mentioned briefly above, I consider that allowing multiple individuals to occupy a patch is more realistic in most cases than restricting occupancy to one individual per patch. In forests, for example, it is not uncommon to find smaller individuals growing below the adult canopy. Even when a species is extremely shade-intolerant, a patch that might hold only one adult can still hold multiple small individuals. The landscape itself in many ways is not realistic but rather represents a best-case scenario: only climate restricts the suitability of patches (e.g. no inappropriate soil types or competition from other species) and the distances to newly suitable climates are shorter than for many latitudinal climate gradients (Loarie *et al.* 2009). Most such complications would be likely to increase lags in range shifts, especially for species with poor dispersal. An exception might be topographical complexity, which can create ‘micro-refugia’ in otherwise unsuitable landscapes. Such refugia might prevent total local extinction at the trailing edge (Franklin *et al.* 2013) or speed up spread if they exist close to the leading range edge (McLachlan *et al.* 2005). Moreover, the 1 × 2 km elevation modelled here would not represent the complete range of most species. In more widely distributed species, latitudinal and elevational shifts might be happening simultaneously, and larger, more complex modelled landscapes could be used to explore the implications of this.

This model, like many others (Atkins and Travis 2010; Bocedi *et al.* 2013; Kubisch *et al.* 2013), uses a single diploid genetic locus governing adaptation to climate. This is an oversimplification, as most traits involved in climate responses are affected by multiple genes (Neale and Savolainen 2004; Weinig *et al.* 2014). It is perhaps best to think of each genotype as a population—for example, that A_1_A_1_ represents a cold-adapted population, A_3_A_3_ a heat-adapted population and A_1_A_3_ an intermediate hybrid. In the case of the plastic species, no evolution could take place because it was represented by a single genotype with a fixed response to the environment. This was done to provide a simple contrast with the initially locally adapted, multi-genotype species. However, plasticity itself is a trait that can evolve, and which can have interesting interactions with local adaption, as other models have explored (e.g. Via and Lande 1985; Tufto 2000; Lande 2009; Gomez-Mestre and Jovani 2013).

The scale of the response curves to the environment was, in these simulations, assumed to be symmetrical for each genotype and the same breadth regardless of whether the species was an annual, a perennial or a tree. This assumption was made in the interest of focusing on the difference between the locally adapted and plastic scenarios. However, it would be worthwhile examining the implications of these assumptions in future studies. While response curves in provenance studies are often fit using symmetrical functions, these may not be ideal in all scenarios (Leites *et al.* 2012; Browne *et al.* 2019; Sebastian-Azcona *et al.* 2019). In terms of the breadth of the response curves, local adaptation has been detected in herbaceous plants over 1000–2000 m elevation gradients (e.g. Halbritter *et al.* 2015). We know that in trees fitness-relevant traits such as onset of cold hardiness, early-life relative growth rate or height at a particular age can, for a particular provenance, vary substantially between sites differing in temperature by 5–10 °C (e.g. Leites *et al.* 2012; Browne *et al.* 2019; Sebastian-Azcona *et al.* 2019). Genetic association analyses have detected differences in allele frequencies linked to environmental gradients in wind-pollinated pines in mountainous environments based on samples taken 5–45 km apart (Eckert *et al.* 2015; Kolb *et al.* 2016). The breadth of each genotype’s tolerance in the locally adapted tree scenarios might therefore be a bit narrow. This would likely serve to increase the apparent difference between the locally adapted and plastic scenarios for this life history category.

In this analysis, the number of alleles was fixed. Other similar models have allowed for mutation, but this might lead to overly optimistic estimates of adaptive potential, as the effect of one new allele in a single-locus model can be quite strong. For instance, Kubisch *et al.* (2013) found that allowing for mutation in the climate response alleles could lead to complete occupation of the landscape. Moreover, adaptation from standing variation is generally considered more likely, especially when generation times are long and the rate of environmental change is rapid (Barrett and Schluter 2008). Some studies have modelled individual traits such as budburst timing with explicit multi-allelic genetic models and examined the effect on range occupancy or fit of traits to the environment (Kramer *et al.* 2010; Kuparinen *et al.* 2010; Cotto *et al.* 2017). In such models, the presence of genetic variation and the possibility of local adaptation also tend to ameliorate, though not eliminate, negative climate change effects on populations.

The results of this simulation suggest that local adaptation in plants tends to decrease lags in tracking climate change if it boosts fitness near the advancing range edge. While local adaptation is common, less is known about how fitness changes across a species range relative to environmental gradients. Several recent analyses have found that, contrary to the ‘abundant centre’ hypothesis, most species examined do not have higher population sizes in the centre of their geographic or climatic ranges, though edge populations may be more patchy on the landscape (Abeli *et al.* 2014; Dallas *et al.* 2017; Pironon *et al.* 2017). Seed production was similar across the range in ~50 % of plants examined (Abeli *et al.* 2014). Over a wide range of species, including plants, Pironon *et al.* (2017) found no consistent centre vs. edge pattern to any vital rates, including survival and fecundity. Few studies have sampled extensively enough to reveal how sharply demographic rates change as one approaches a range boundary. Some exceptions include Jump and Woodward (2003), Samis and Eckert (2007), Vaupel and Mattheis (2012) and Moeller *et al.* (2012). Of the demographic rates examined, many show no pattern while those that do often show linear declines with some measure of ‘marginality’. However, it would be useful to re-analyse such data sets in terms of environmental distance rather than physical distance.

Finally, are the pollen and seed dispersal kernels used in the model realistic? The seed dispersal kernels were inspired by a previous spread analysis based on actual species traits (Lustenhower *et al.* 2017). As that paper noted, there are multiple ways that seed dispersal has been measured, not all of which are directly comparable, and many plant species lack accessible data on this and other relevant parameters. Pollen dispersal distances are even less reported on. While this analysis held pollen dispersal for a life history class constant, there is likely variation in pollen dispersal within such groups. Both can also vary due to changes in biotic or abiotic dispersal vectors. For instance, pollinator behaviour can be one of the factors limiting plant reproduction at range boundaries (Moeller *et al.* 2012). However, all else being equal, longer seed dispersal distances should aid species in tracking climate shifts, while very limited dispersal will hamper them. The effects of pollen dispersal are likely to be more complicated, as extensive pollen movement may hamper local adaptation and would not directly aid spread, since pollen cannot ‘outrun’ the leading edge of the population.

Based on the results of these simulations and the unknowns outlined above, I would propose several avenues of research that may aid in predicting how well plant species will track climate change, and which may be at risk.

1) Examination of local adaptation in annual species that have poor dispersal and/or highly fragmented ranges, and comparison of observed range shifts in such species to co-occurring species with more effective dispersal mechanisms.
2) Comparison of predicted shifts in climate gradients to generation time and average dispersal distances of tree species. Species in which the predicted climate shift over a generation is greater than 10 times the average dispersal distance may be most at risk, and could be targeted for further study.
3) Simulation analyses of predicted spread rates based on trait values through actual landscapes—including patchy habitat suitability and competition—that can be compared to observed shifts (such as those documented in Lenoir *et al.* (2008) or Beckage *et al.* (2008)). This would help identify the most important factors that interact with dispersal, time to maturity and local adaptation to speed up or slow down shifts.

## Supporting Information

The following additional information is available in the online version of this article—

**Appendix S1.** Model details.

**Appendix S2.** Additional model results.

plaa008_suppl_Supplementary_Material

## Data

Model code is available on GitHub: https://github.com/emoran5/LocalAdaptation_RangeShifts

## Sources of Funding

Models were run on the MERCED computer cluster at the University of California Merced (supported by NSF Award ACI-1429783).

## Conflict of Interest

None declared.
