# Supplementary material for: Simulating the effects of local adaptation and life history on the ability of plants to track climate shifts
Source: AoB Plants. 2020 Feb 14;12(1):plaa008. doi: 10.1093/aobpla/plaa008 (PMC7046178; doi:10.1093/aobpla/plaa008)
Supplement: plaa008_suppl_Supplementary_Material [file plaa008_suppl_supplementary_material.pdf]

Appendix S1: Model details

Population initiation

Table S1.1: Initial number of individuals of each genotype placed in each climate zone. For plastic single-genotype scenarios, the number of individuals equals the total number for the genetically-diverse scenario

**Climates:**	**10**	**9**	**8**	**7**	**6**	**5**	**4**	**3**	**2**
**Trees (saplings)**									
A_1A_1					10	30	100	50	30
A_1A_2			10	20	40	100	60	26	10
A_2A_2		2	15	25	45	25	15	2	
A_1A_3		2	15	25	45	25	15	2	
A_2A_3	10	26	50	100	50	20	10		
A_3A_3	30	50	100	30	10				
Total	*40*	*80*	*200*	*200*	*200*	*200*	*200*	*80*	*40*
**Annuals (seeds) and perennials (small adults)**									
A_1A_1					100	300	1000	500	300
A_1A_2			100	200	400	1000	600	260	100
A_2A_2		20	150	250	450	250	150	20	
A_1A_3		20	150	250	450	250	150	20	
A_2A_3	100	260	500	1000	500	200	100		
A_3A_3	300	500	1000	300	100				
Total	*400*	*800*	*2000*	*2000*	*2000*	*2000*	*2000*	*800*	*400*

Dispersal probabilities

The steps to calculate the probability of dispersing seed or pollen from a given landscape to patch to any other were as follows:

1) Identify the dispersal parameter that would yield the correct mean dispersal. Because the equation for the 2D-t dispersal kernel is

$$prob.disp = \frac{1}{\left(\pi u \left(1 + \frac{d^2}{u}\right)^2\right)}$$

where d is the dispersal distance, this is given by:

$$u = (mean.disp \times 2/\pi)^2$$

2) Calculate probability of dispersing to a range of distances from 0 to 2010 m, at 0.1 m intervals using 2D-t kernel.

3) Add up probabilities within 20 m categories, and divide by the total sum over all distances to ensure probabilities add up to 1.

4) Calculate the number of patches within 20 m distance categories to a source patch.

5) Within each distance category, divide total dispersal probability by number of patches.

6) Calculate a distance matrix showing the distance between the center of each patch in the landscape and the center of every other patch.

7) Using this distance matrix, fill in a dispersal probability matrix of dimensions (number of patches) x (number of patches).

The results for dispersal from a single source patch for two different mean dispersal distances (probabilities multiplied by 5000 seeds to show expected number of seeds dispersing to each patch) is shown in Figure S1.1 below.

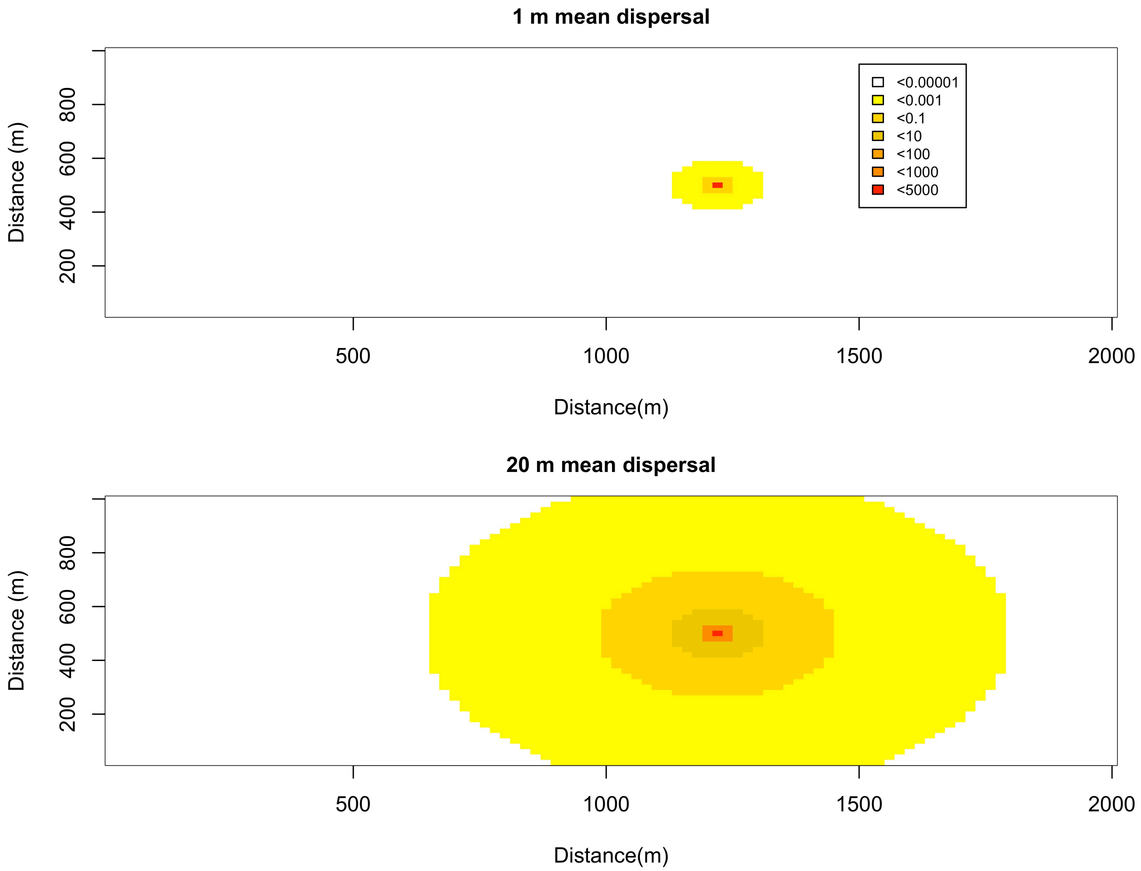

Figure S1.1: Average number of seeds out of a crop of 5000 dispersing from a source square (in red) to other squares in the landscape for species with an average dispersal distance of 1 m (top) vs. 20 m (bottom).

For the plastic species, because all individuals have the same genotype we need only calculate the numbers of seeds traveling different distances and can ignore pollen dispersal. For the locally adapted species, we must first calculate how much pollen carrying which alleles will be received by each patch. The ovules produced in each patch carry one of the two alleles of the mother plant, with equal probability. For example, the number of ovules in a patch carrying allele A_1 is equal to the total seed production of A_1A_1 individuals plus half the total seed production of A_1A_2 and A_1A_3 individuals. The probability that an A_1 ovule will result in an A_1A_1 seed is equal to the proportion of the pollen arriving in the patch that carries the A_1 allele, and likewise for all other seed genotypes. The numbers of seeds of each

genotype are calculated, the seeds are dispersed according to the probabilities explained above.

Climate effects:

Table S1.1: Climate effects on fecundity and/or survival probabilities

Scenario	Optimum	± 1	± 2	± 3	± 4	± 5	± ≥6
Locally adapted: A_1A_1 & A_3A_3	0.9 (1 band each)	0.5	0.3	0.15	0	0	0
Locally adapted: A_1A_2, A_2A_2, A_1A_3, & A_2A_3	1 (1 band each)	0.7	0.4	0.25	0.1	0	0
LA perennial, weak climate effects: A_1A_1 & A_3A_3	0.95 (1 band each)	0.75	0.65	0.575	0.25	0	0
LA perennial, weak climate effects: A_1A_2, A_2A_2, A_1A_3, & A_2A_3	1 (1 band each)	0.85	0.7	0.625	0.55	0.25	0
Standard plastic	1 (1 band)	0.9	0.75	0.4	0.1	0	0
Widely plastic	1 (3 bands)	0.7	0.5	0.2	0.1	0	0

Table S1.2: Climate effects on germination and/or transition probabilities

Scenario	Optimum	± 1	± 2	± 3	± 4	± 5	± ≥6
Locally adapted	1 (1 band/genotype)	0.7	0.5	0.2	0.1	0	0
LA perennial, weak climate effects	1 (1 band/genotype)	0.85	0.75	0.6	0.55	0.25	0
Standard plastic	1 (1 band)	0.85	0.7	0.4	0.2	0	0
Widely plastic	1 (4 bands)	0.7	0.5	0.2	0.1	0	0

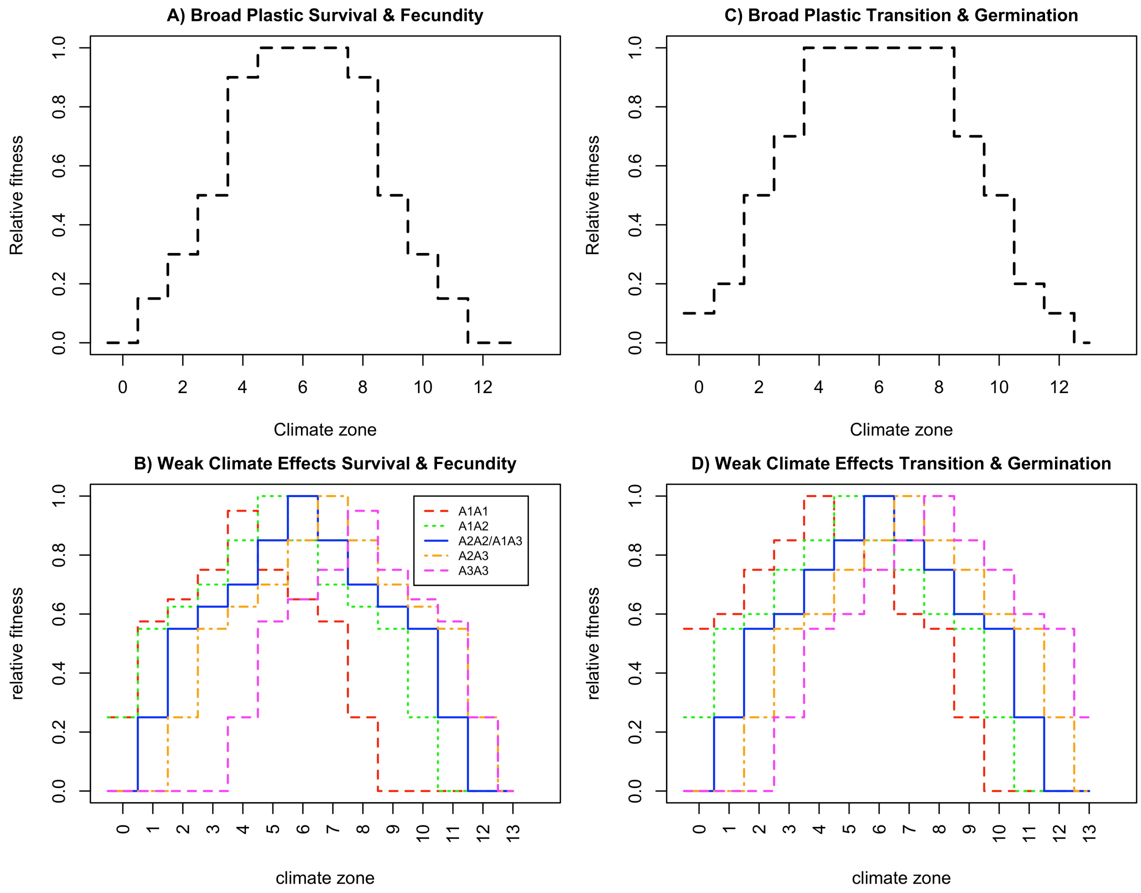

Figure S1.2: Fitness of broadly plastic perennial species across climates in terms of survival and fecundity (A) and in terms of transition and germination (C). Fitness of perennial with weakened climatic responses in terms of survival and fecundity (B) and in terms of transition and germination (D).

Appendix S2: Additional model results

Table S2.1: Percent change in landscape occupancy at the end of the climate change period ("Shift") and at the end of the restabilization period ("Restab"). MD indicates mean seed dispersal (m). Standard deviation over 4 runs given in brackets. Numbers in italics indicate range loss due to loss of suitable habitats from landscape. An asterix indicates losses of over 5% not due to loss of suitable habitat. Example range shift maps for scenarios shown in red are given in Figures S2.1 through S2.21.

		**% change to original range**			
		**Locally adapted**		**Plastic**	
**Life-form**	**MD**	**Shift**	**Restab**	**Shift**	**Restab**
Annual, standard	1	*-5.84 [0.18]*	*-11.08 [0.09]*	-27.75* [0.14]	20 yr: -29.29* [0.16]<br>200 yr: -33.89*
	20	*-7.33 [0.1]*	*-10.36 [0.21]*	-7.87* [0.71]	-0.15 [0.13]
	50	*-9.95 [0.01]*	*-10.02 [0.01]*	-0.09 [0.17]	-0.26 [0.22]
Perennial, standard	20	0.74 [0.63]	0.79 [0.38]	-9.25* [1.27]	-0.07 [0.35]
	80	0.15 [0.26]	*-3.20 [0.38]*	-4.16 [0.26]	-0.29 [0.32]
Perennial, weaker climate effects	20	*-2.07 [0.44]*	*-10.84 [0.25]*	N/A	N/A
Perennial, wider tolerance	20	N/A	N/A	0.08 [0.45]	*-7.74* [0.52]
Tree, standard	40	-2.49	-0.27	-16.13*	-0.05

		[0.84]	[0.43]	[0.16]	[0.32]
	80	-4.02<br>[0.34]	-0.17<br>[0.53]	-8.32*<br>[0.63]	-0.2<br>[0.53]
Tree, 2x shift rate, standard landscape	40	-43.21*<br>[1.88]	*-23.62*<br>*[0.33]*	N/A	N/A
	80	-31.46*<br>[1.48]	*-25.56*<br>*[0.19]*	N/A	N/A
Tree, 2x shift rate, extended landscape	40	-42.42*<br>[0.84]	-0.2<br>[0.49]	-32.36*<br>[0.45]	0.28<br>[0.17]
	80	-31.94*<br>[1.16]	-0.09<br>[0.31]	-19.97*<br>[0.64]	-0.08<br>[0.25]
Tree, 4x wider climate bands	40	-15.34*<br>[0.42]	0.15<br>[0.13]	-27.07*<br>[0.27]	0.01<br>[0.05]
	80	-8.7*<br>[0.58]	-0.07<br>[0.16]	-22.77*<br>[0.12]	0.02<br>[0.12]

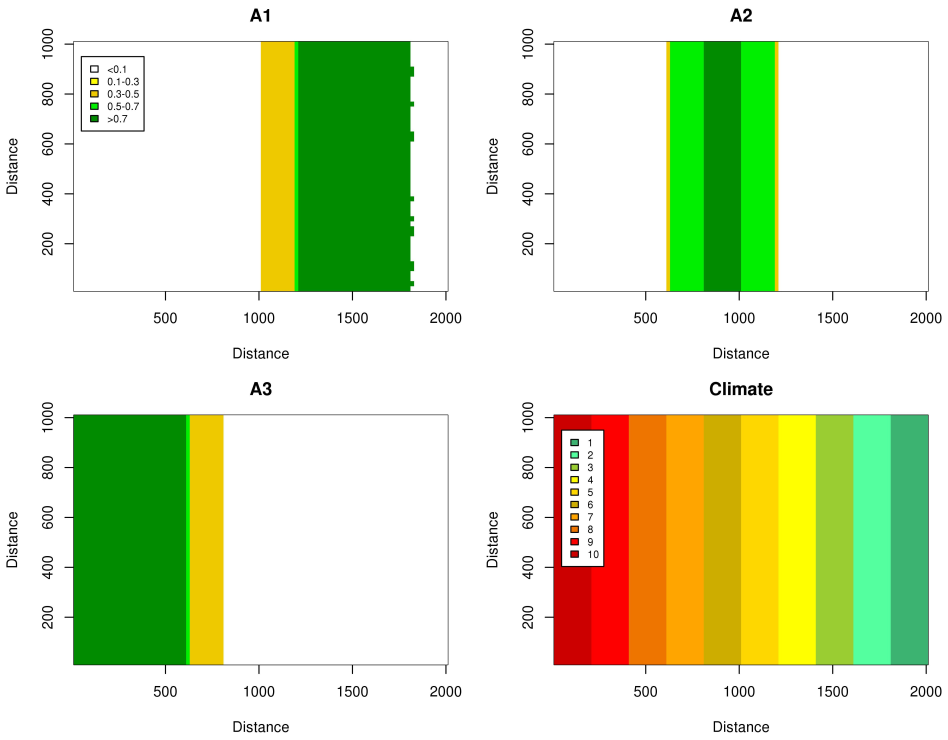

Figure S2.1: Locally adapted annual with 1 m average seed dispersal after 20 year initialization period. Distribution of each allele indicated, with colors indicating allele frequencies from 10-30% (light yellow) to >70% (dark green). Fourth panel indicates initial distribution of climates.

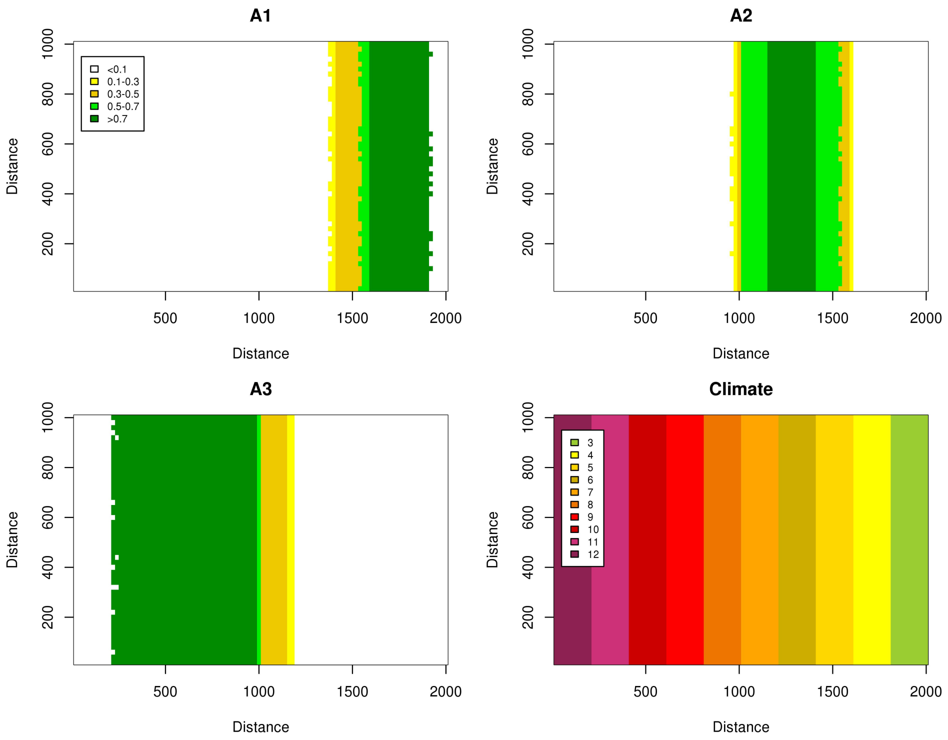

Figure S2.2: Locally adapted annual with 1 m average seed dispersal after 20 years of climate change. Distribution of each allele indicated, with colors indicating allele frequencies from 10-30% (light yellow) to >70% (dark green). Fourth panel indicates final distribution of climates.

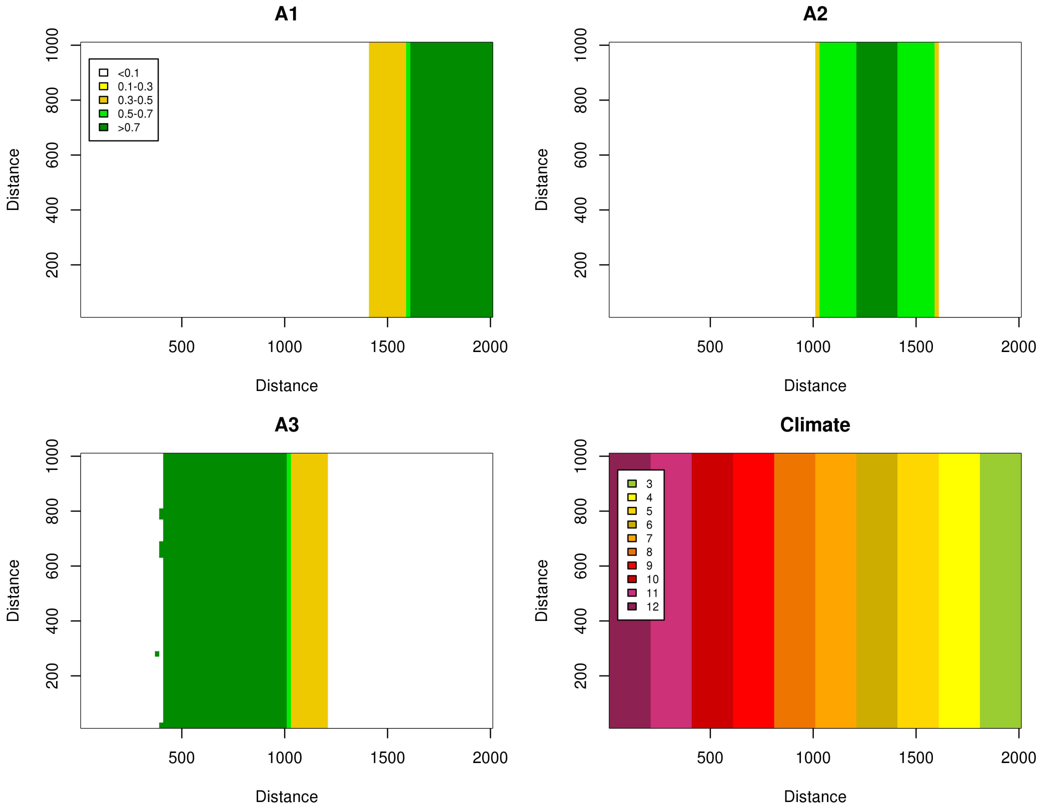

Figure S2.3: Locally adapted annual with 1 m average seed dispersal after 20 years of restabilization following climate change. Distribution of each allele indicated, with colors indicating allele frequencies from 10-30% (light yellow) to >70% (dark green). Fourth panel indicates final distribution of climates.

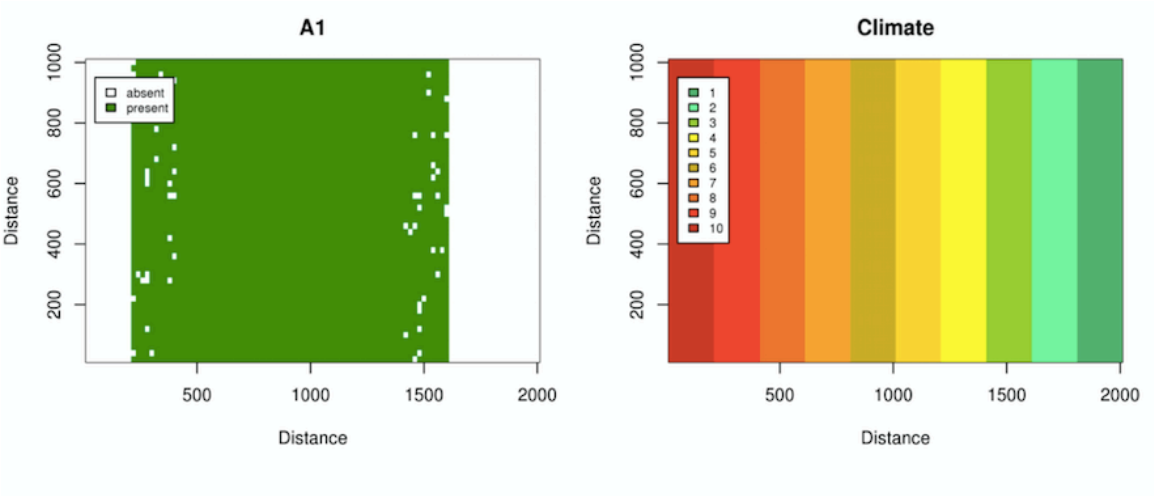

Figure S2.4: Plastic annual with 1 m average seed dispersal after 20 year initialization period. Green indicates occupied patch. Second panel indicates initial distribution of climates.

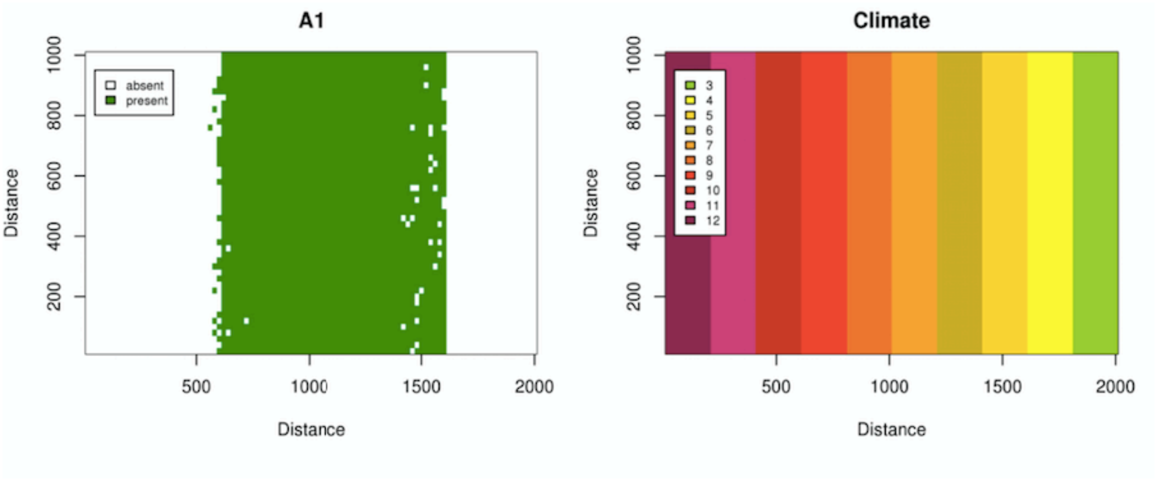

Figure S2.5: Plastic annual with 1 m average seed dispersal after 20 years of climate change. Green indicates occupied patch. Second panel indicates final distribution of climates.

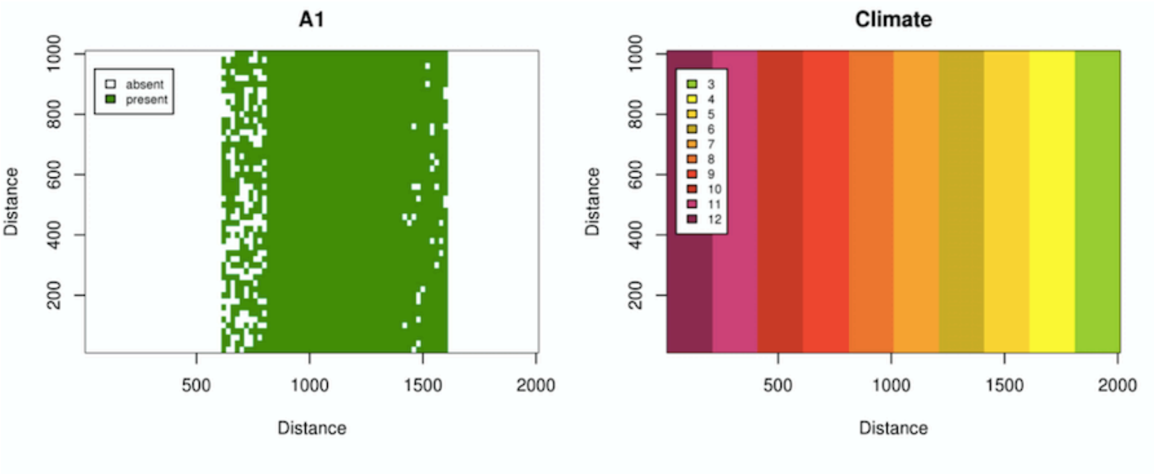

Figure S2.6: Plastic annual with 1 m average seed dispersal after 200 years of restabilization. Green indicates occupied patch. Second panel indicates final distribution of climates.

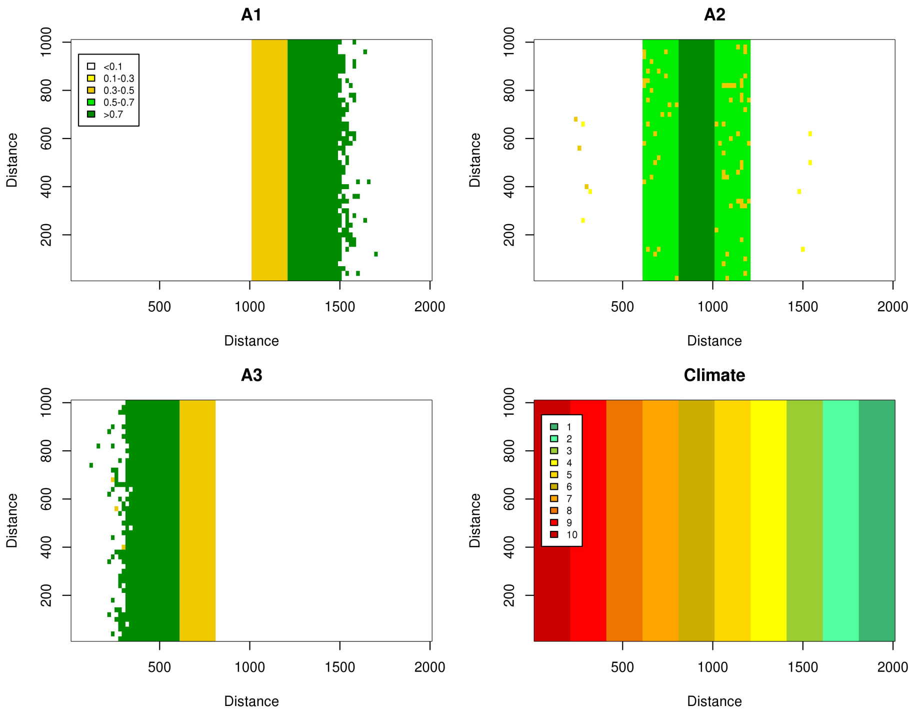

Figure S2.7: Locally adapted perennial with 20 m average seed dispersal after 100 year initialization period. Distribution of each allele indicated, with colors indicating allele frequencies from 10-30% (light yellow) to >70% (dark green). Fourth panel indicates initial distribution of climates.

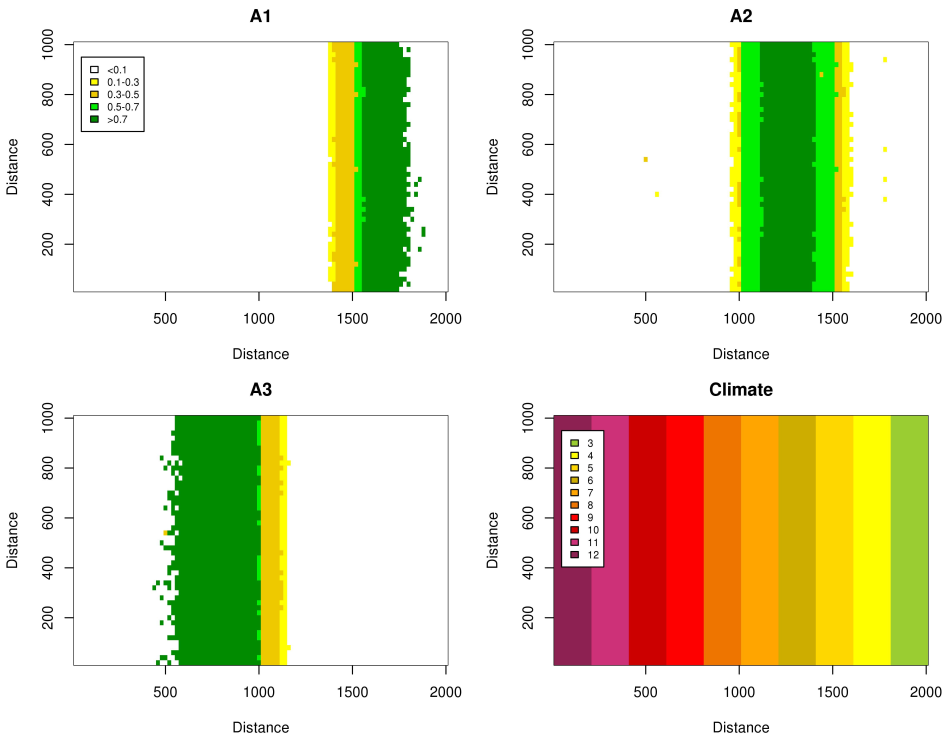

Figure S2.8: Locally adapted perennial with 20 m average seed dispersal after 20 years of climate change. Distribution of each allele indicated, with colors indicating allele frequencies from 10-30% (light yellow) to >70% (dark green). Fourth panel indicates final distribution of climates.

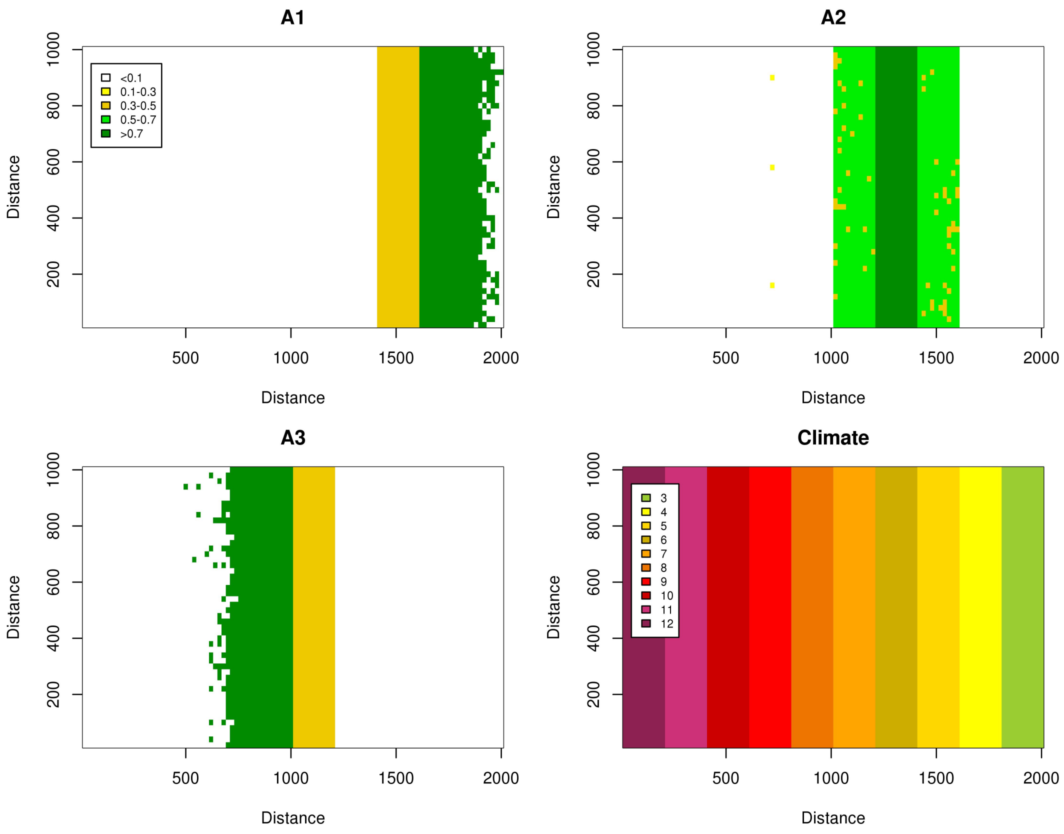

Figure S2.9: Locally adapted perennial with 20 m average seed dispersal after 100 years of restabilization. Distribution of each allele indicated, with colors indicating allele frequencies from 10-30% (light yellow) to >70% (dark green). Fourth panel indicates final distribution of climates.

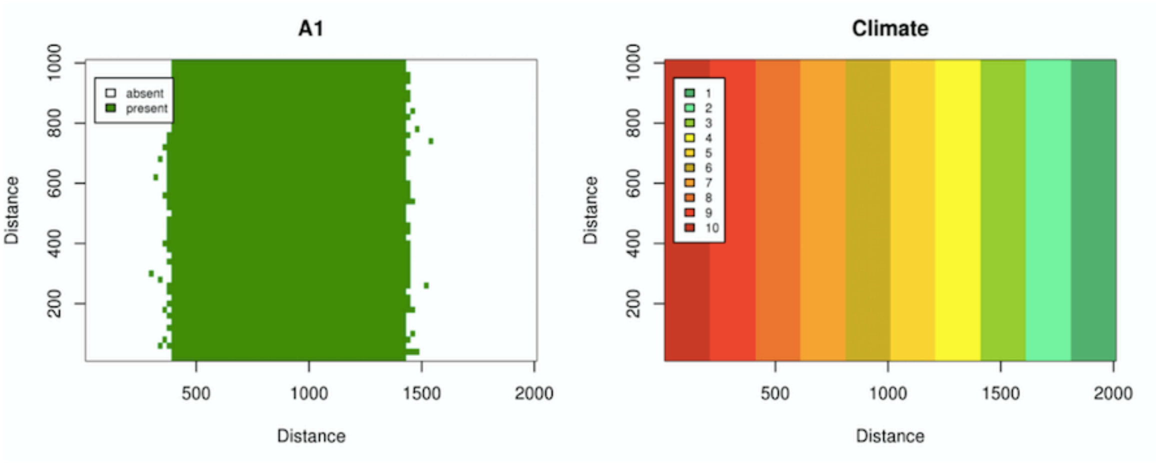

Figure S2.10: Plastic perennial with 20 m average seed dispersal after 100 year initialization period. Green indicates occupied patches. Second panel indicates initial distribution of climates.

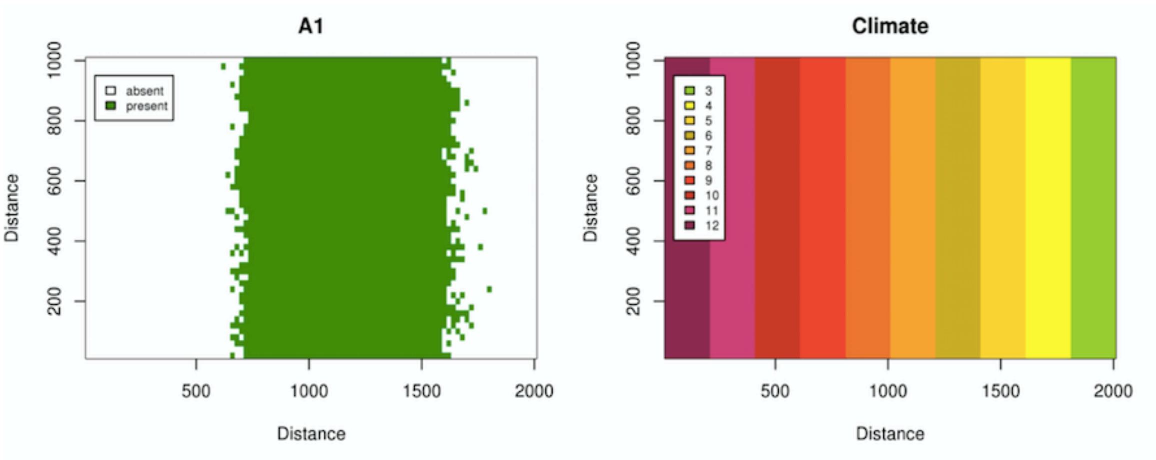

Figure S2.11: Plastic perennial with 20 m average seed dispersal after 20 years of climate change. Green indicates occupied patches. Second panel indicates final distribution of climates.

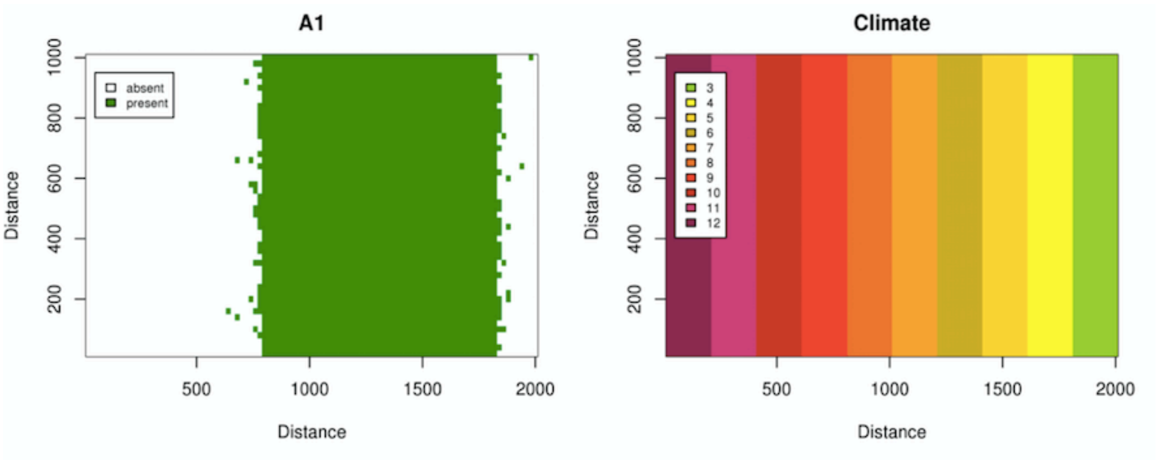

Figure S2.12: Plastic perennial with 20 m average seed dispersal after 100 years of restabilization. Green indicates occupied patches. Second panel indicates final distribution of climates.

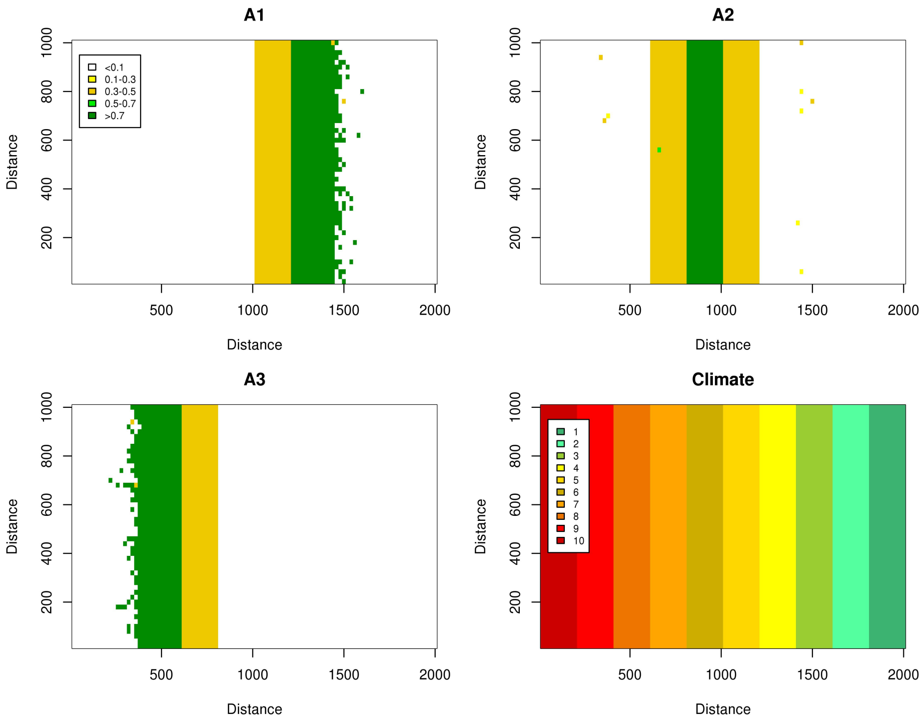

Figure S2.13: Locally adapted tree with 40 m average seed dispersal after 200 year initialization period. Distribution of each allele indicated, with colors indicating allele frequencies from 10-30% (light yellow) to >70% (dark green). Fourth panel indicates initial distribution of climates.

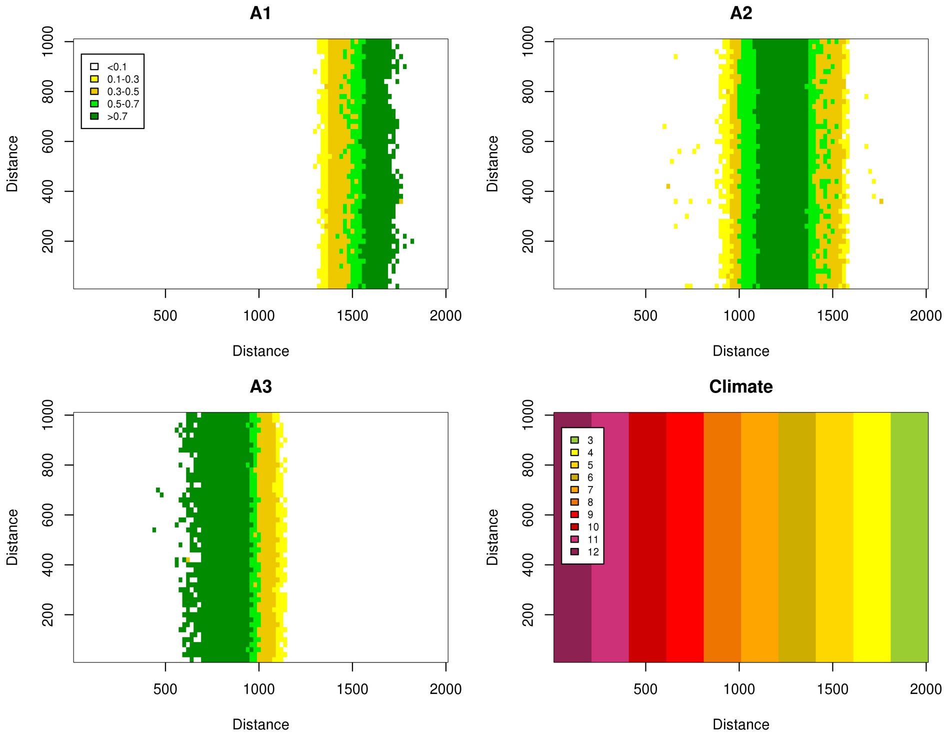

Figure S2.14: Locally adapted tree with 40 m average seed dispersal after 20 years of climate change. Distribution of each allele indicated, with colors indicating allele frequencies from 10-30% (light yellow) to >70% (dark green). Fourth panel indicates initial distribution of climates.

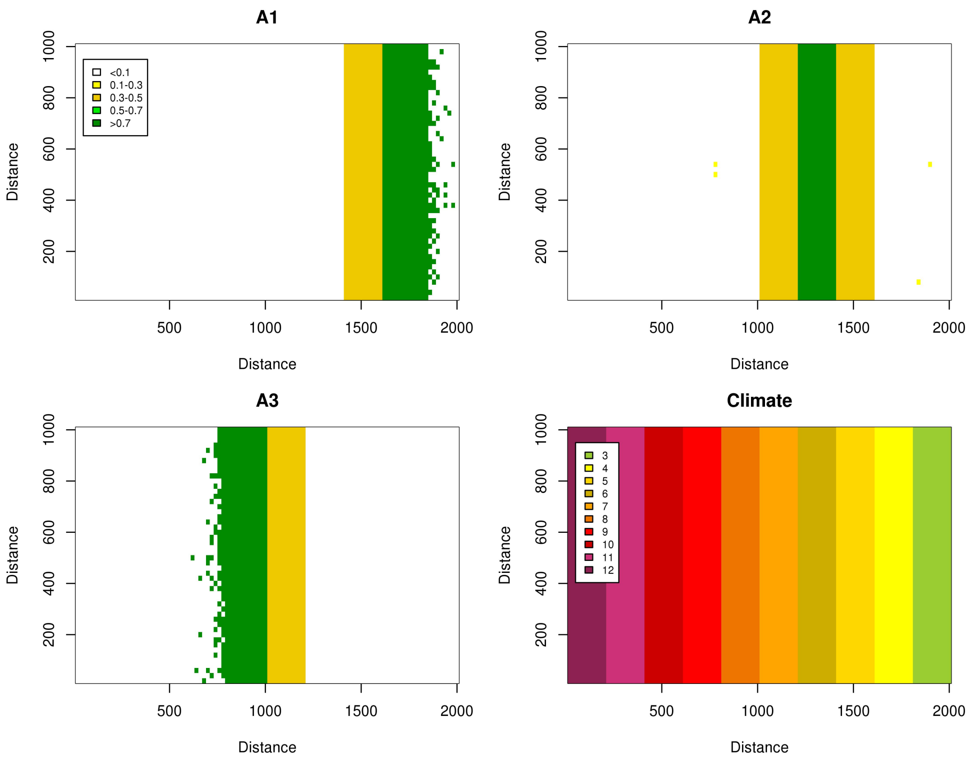

Figure S2.15: Locally adapted tree with 40 m average seed dispersal after 200 years of restabilization. Distribution of each allele indicated, with colors indicating allele frequencies from 10-30% (light yellow) to >70% (dark green). Fourth panel indicates final distribution of climates.

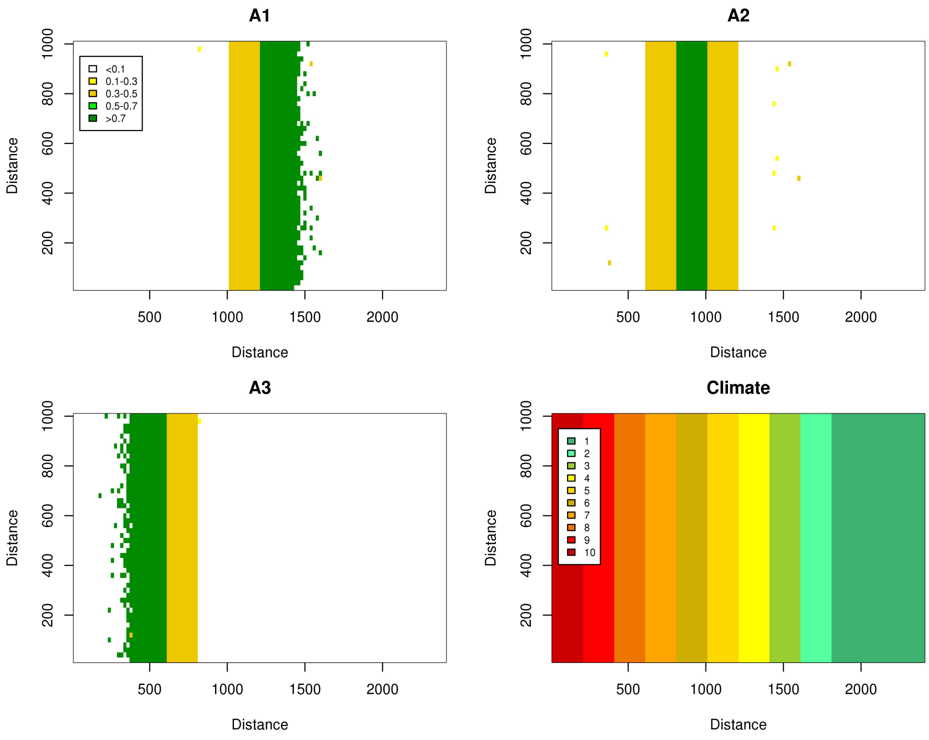

Figure S2.16: Locally adapted tree with 40 m average seed dispersal after 200 year initialization period on landscape with extra cold-climate bands. Distribution of each allele indicated, with colors indicating allele frequencies from 10-30% (light yellow) to >70% (dark green). Fourth panel indicates initial distribution of climates.

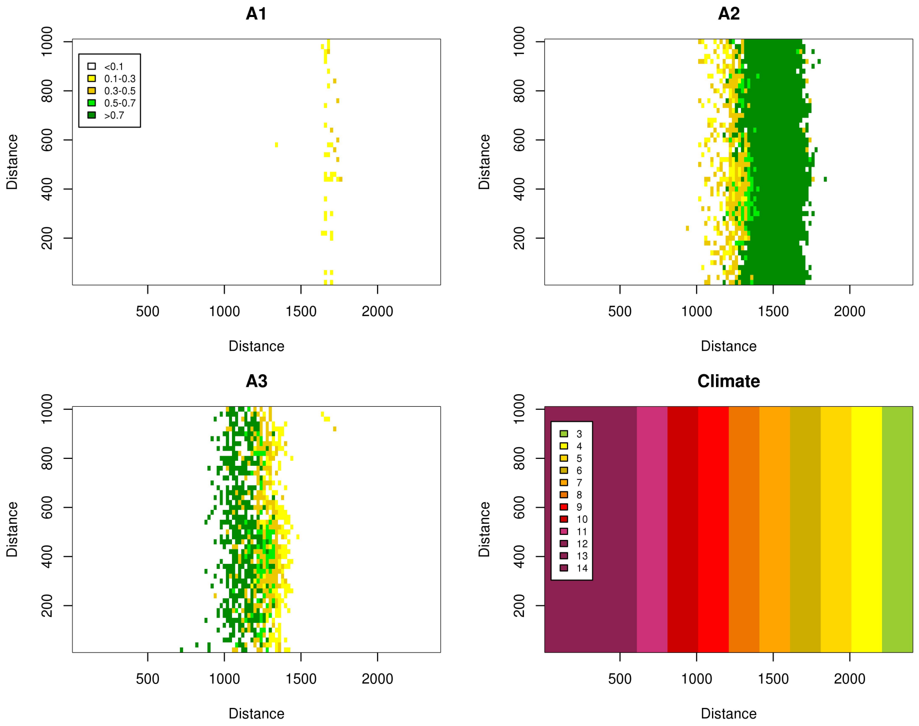

Figure S2.17: Locally adapted tree with 40 m average seed dispersal after 20 years of 2x faster climate change. Distribution of each allele indicated, with colors indicating allele frequencies from 10-30% (light yellow) to >70% (dark green). Fourth panel indicates final distribution of climates.

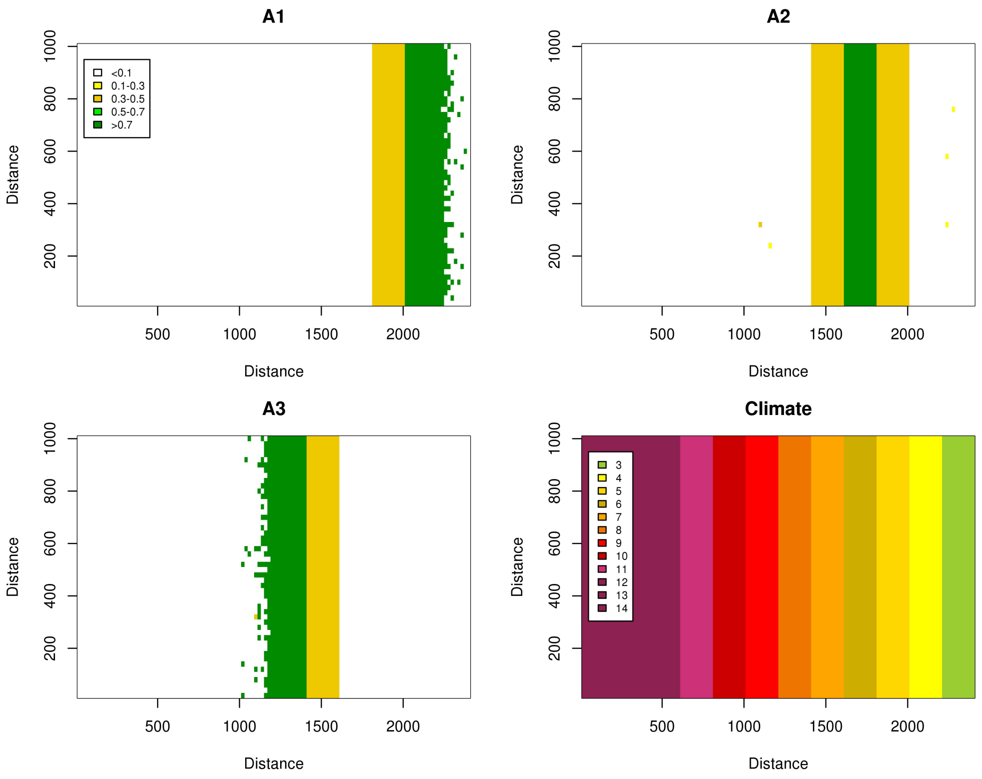

Figure S2.18: Locally adapted tree with 40 m average seed dispersal after 200 years of restabilization after rapid climate shift. Distribution of each allele indicated, with colors indicating allele frequencies from 10-30% (light yellow) to >70% (dark green). Fourth panel indicates final distribution of climates.

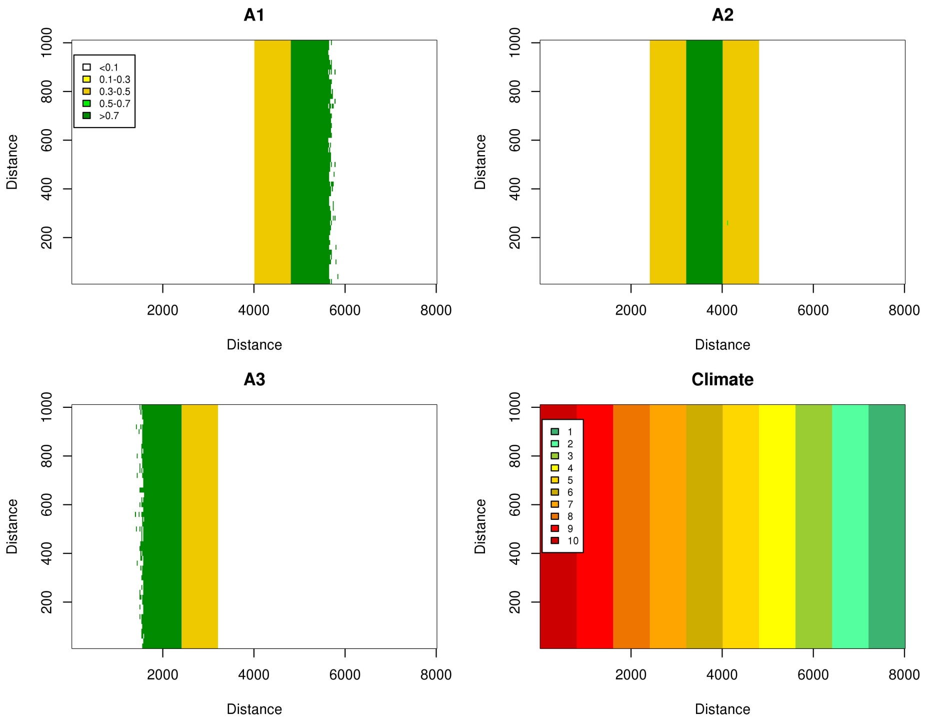

Figure S2.19: Locally adapted tree with 40 m average seed dispersal after 200 year initialization period on shallow-gradient landscape (climate bands 4x wider). Distribution of each allele indicated, with colors indicating allele frequencies from 10-30% (light yellow) to >70% (dark green). Fourth panel indicates initial distribution of climates.

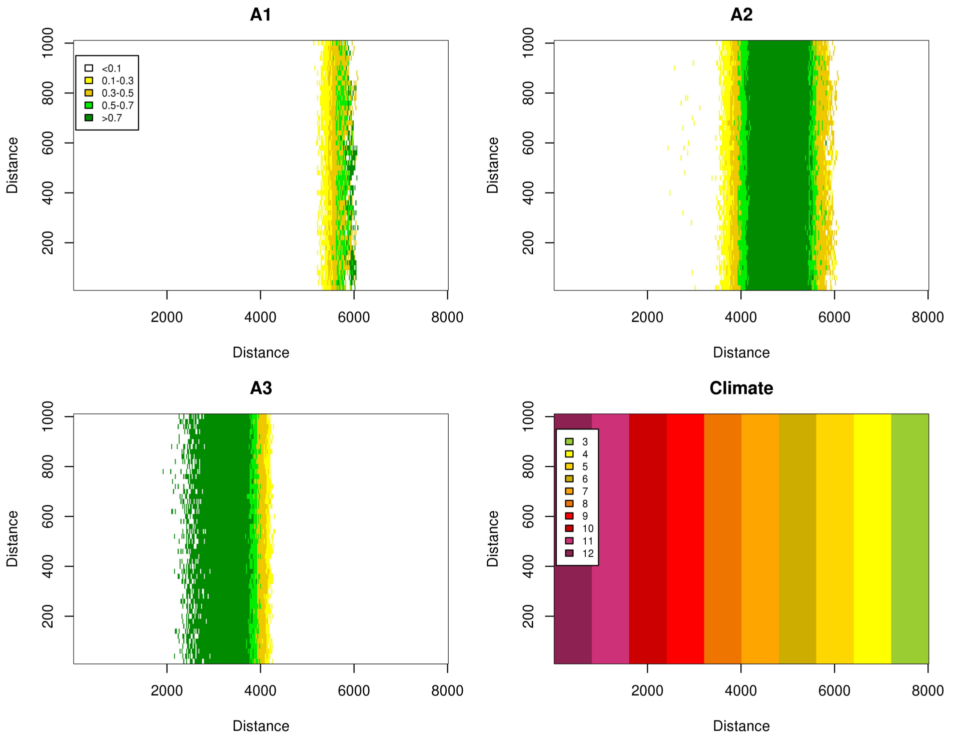

Figure S2.20: Locally adapted tree with 40 m average seed dispersal after 20 years of climate change on shallow-gradient landscape. Distribution of each allele indicated, with colors indicating allele frequencies from 10-30% (light yellow) to >70% (dark green). Fourth panel indicates final distribution of climates.

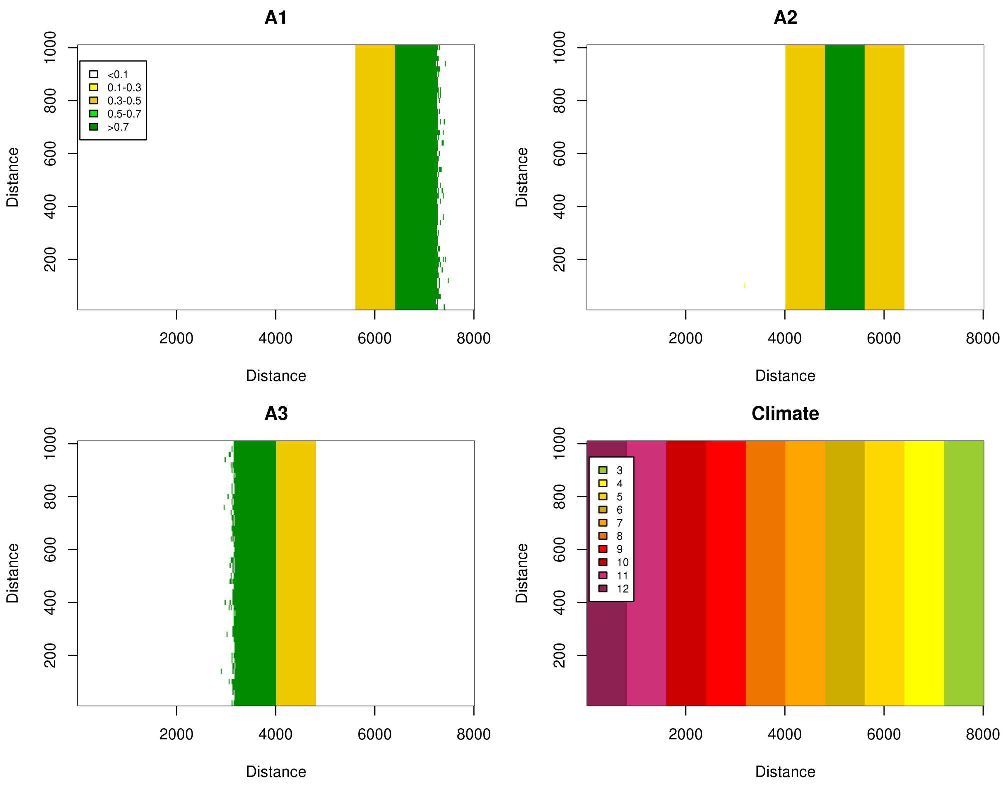

Figure S2.21: Locally adapted tree with 40 m average seed dispersal after 200 years of restabilization on shallow-gradient landscape. Distribution of each allele indicated, with colors indicating allele frequencies from 10-30% (light yellow) to >70% (dark green). Fourth panel indicates final distribution of climates.
